# Programmable Electro‐Assembly of Collagen: Constructing Porous Janus Films with Customized Dual Signals for Immunomodulation and Tissue Regeneration in Periodontitis Treatment

**DOI:** 10.1002/advs.202305756

**Published:** 2024-01-08

**Authors:** Miao Lei, Haoran Wan, Jia Song, Yanhui Lu, Ronghang Chang, Honglei Wang, Hang Zhou, Xuehui Zhang, Changsheng Liu, Xue Qu

**Affiliations:** ^1^ Key Laboratory for Ultrafine Materials of Ministry of Education Frontiers Science Center for Materiobiology and Dynamic Chemistry School of materials science and engineering East China University of Science and Technology Shanghai 200237 China; ^2^ Department of Dental Materials & Dental Medical Devices Testing Center NMPA Key Laboratory for Dental Materials Peking University School and Hospital of Stomatology Beijing 100081 China; ^3^ Shanghai Frontier Science Research Base of Optogenetic Techniques for Cell Metabolism East China University of Science and Technology Shanghai 200237 China; ^4^ Wenzhou Institute of Shanghai University Wenzhou 325000 China

**Keywords:** collagen, electro‐assembly, immunomodulatory activity, Janus porous structure, periodontitis treatment

## Abstract

Currently available guided bone regeneration (GBR) films lack active immunomodulation and sufficient osteogenic ability‐ in the treatment of periodontitis, leading to unsatisfactory treatment outcomes. Challenges remain in developing simple, rapid, and programmable manufacturing methods for constructing bioactive GBR films with tailored biofunctional compositions and microstructures. Herein, the controlled electroassembly of collagen under the salt effect is reported, which enables the construction of porous films with precisely tunable porous structures (i.e., porosity and pore size). In particular, bioactive salt species such as the anti‐inflammatory drug diclofenac sodium (DS) can induce and customize porous structures while enabling the loading of bioactive salts and their gradual release. Sequential electro‐assembly under pre‐programmed salt conditions enables the manufacture of a Janus composite film with a dense and DS‐containing porous layer capable of multiple functions in periodontitis treatment, which provides mechanical support, guides fibrous tissue growth, and acts as a barrier preventing its penetration into bone defects. The DS‐containing porous layer delivers dual bio‐signals through its morphology and the released DS, inhibiting inflammation and promoting osteogenesis. Overall, this study demonstrates the potential of electrofabrication as a customized manufacturing platform for the programmable assembly of collagen for tailored functions to adapt to specific needs in regenerative medicine.

## Graphical abstract

A drug‐modulated electro‐assembly of collagen GBR film, offering customized porous microstructures (i.e., porosity and pore size) and therapeutic salt component is reported here. This water‐based fabrication method is convenient, fast, and allows stepwise creation of bioactive GBR film with dense/porous Janus layers. The coupling of microstructures and specific anti‐inflammatory components within the Janus film demonstrates a synergistic potential for inflammation suppression and alveolar bone regeneration promotion.

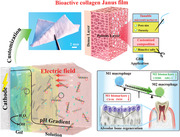

## 1 Introduction

Periodontitis represents a prevalent chronic inflammatory disease. Persistent inflammation of periodontal tissues leads to severe destruction of the supporting structures, including the alveolar bone (AB), periodontal ligament, and cementum, eventually resulting in tooth loss.^[^ ^1^ ^]^ The primary treatment goals involve mitigating inflammation and fostering the regeneration of functional periodontal tissues, with AB reconstruction being a pivotal step in this process.^[^ ^2^ ^]^ In clinical practice, GBR is an effective approach for reconstructing AB. This technique involves the application of a barrier film, known as a GBR film, to impede the rapid growth of gingival connective tissue and thwart its invasion into bone defects.^[^ ^3^ ^]^ Presently, tissue‐derived BioGide films represent the most widely used commercially available absorbable GBR films, distinguished by their remarkable compatibility owing to the predominant collagen matrix. However, these films primarily function as post‐implantation barriers and possess limited biological activity in terms of their anti‐inflammatory and osteogenic properties.^[^ ^4^ ^]^ Ideal GBR films should exhibit anisotropic physicochemical attributes at the interface between the gingival connective tissue and AB, conferring differential biological functions.^[^ ^5^ ^]^ Specifically, the film surface facing the gingival tissue should have a dense structure, ensuring robust mechanical support to prevent film collapse and acting as a barrier against fibroblast invasion, thus providing a stable space and ample time for bone regeneration. In contrast, the film surface facing the bone defect should demonstrate enhanced “bioactivity”, fostering an optimal physiological microenvironment (ME) conducive to osteogenesis‐related cells amidst the periodontal inflammatory milieu and thereby promoting efficient bone regeneration.

Osteogenesis is a multifaceted process governed by various cell types, with particular emphasis on the interplay between osteoblasts and inflammatory cells (e.g., macrophages, the most abundant inflammatory cells).^[^ ^6^ ^]^ Diverse phenotypes have distinct effects on osteogenesis. In the context of periodontal inflammation, the imbalance between the pro‐inflammatory M1 and anti‐inflammatory M2 phenotypes of macrophages (i.e., an excessive M1 phenotype of macrophages) becomes a critical hindrance to AB regeneration.^[^ ^4^, ^7^ ^]^ Therefore, active regulation of the immune ME following GBR film implantation becomes crucial in periodontal inflammation treatment. The surface topology and releasable components of the implanted materials provide relevant “physical” and “molecular” cues that initiate subsequent cellular responses.^[^ ^8^ ^]^ Porous structures have been identified as significant physical cues that influence macrophage phenotypes and host immune responses.^[^ ^9^ ^]^ In comparison, non‐porous structures on similar matrix materials induce an M1 phenotype in macrophages, whereas porous structures promote their transition towards an M2 phenotype.^[^ ^10^ ^]^ Moreover, a larger pore size and increased porosity further enhance macrophage polarization towards the M2 phenotype, leading to the downregulation of inflammatory cytokines and upregulation of anti‐inflammatory cytokines.^[^ ^11^ ^]^ The implanted materials regulate the macrophage phenotype by releasing functional components (e.g., interleukin‐4 (IL‐4) and active metal ions) and are another effective means for constructing an anti‐inflammatory immune ME.^[^ ^12^ ^]^ Hence, by strategically designing the surface topology and composition of the GBR films, they can be endowed with bioactive immunomodulatory functionality, thereby facilitating bone regeneration in an inflammatory context for the treatment of periodontitis.

Electrofabrication is an emerging additive manufacturing approach that uses imposed electrical signals (generally <5 V) to guide the assembly of biopolymers on (or near) electrode surfaces.^[^ ^13^ ^]^ The strengths of electrofabrication are that it is generally simple, rapid (minutes), reagentless, and offers unprecedented capabilities for controlling the structure and function of materials.^[^ ^14^ ^]^ In a previous study, electrical signals were employed to assemble biomacromolecular films on electrode surfaces by controlling both the microstructure and macroscopic geometries of these assemblies.^[^ ^15^ ^]^ This precise hierarchical supramolecular structure arises from charged macromolecules responding to the electrostatic field induced by electrical signals and the subtle tuning of intermolecular interactions owing to the locally high pH from electrochemical reactions. Additionally, the electrostatic shielding of ions significantly alters the response of charged polymers to the electrostatic field, affecting the pH‐dependent balance of interchain interactions, kinetic assembly of polymers, and organization of polymers.^[^ ^16^ ^]^

**Scheme 1:**
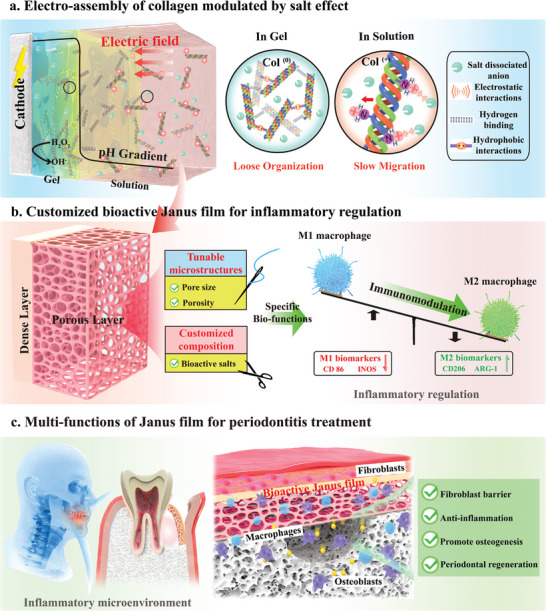
a) Illustration of how soluble salts modulate the electro‐assembly of collagen and the potential mechanism. Anions dissociated from soluble salts screen the collagen's positive charge in solution through electrostatic interactions, resulting in the slow migration of collagen molecules to the cathode under the applied electric field, then assemble into a loosely organized (i.e., high water content) hydrogel network at the cathode surface due to the locally high pH generated by the cathode reaction. b) Sequential two‐step electro‐assembly of collagen using the solutions with pre‐programmable ionic environments (i.e., salt species, concentration), allows customization of a collagen‐based Janus porous film with specific porous microstructures (i.e., porosity and pore size) and bioactive salt composition (i.e., anti‐inflammatory drug, DS) that endow the bio‐functions of inflammatory regulation. c) The coupling of specific microstructures and anti‐inflammatory composition of Janus film can provide asymmetric cellular responses (preventing fibroblast infiltration but promoting osteoblast growth, and differentiation) and immunomodulatory functions, thereby inhibiting inflammation and promoting periodontal tissue regeneration.

In this study, we expanded the electroassembly of collagen in programmable ionic environments to fabricate collagen films with a precise porous topology, encompassing specific porosity and pore size. This approach uses ionic drugs with anti‐inflammatory properties, such as DS. The strategic integration of DS within the collagen film allows for loading and controlled DS release, rendering the film not only an efficient carrier of DS but also a platform for maintaining its inherent porous structure. Furthermore, we demonstrated that the sequential electro‐assembly of collagen under pre‐programmed DS environmental conditions could construct Janus porous films with dense/porous asymmetric structures containing DS, serving multiple functions for periodontitis treatment. First) the dense Janus film layer acts as a guide for the growth of connective tissue fibroblasts, functioning as a barrier to prevent their invasion into bone defects. ii) the porous Janus film layer exhibits immunoregulatory activity through the combined effects of its porous topology and the sustained release of DS “molecular signals.” This leads to the inhibition of the M1 phenotype of macrophages and promotes the M2 phenotype under inflammatory conditions, indirectly contributing to the regeneration of AB in periodontitis, as illustrated in **Scheme** **1**. Our study highlights the potential of using the electrostatic shielding effect to precisely control the electroassembly of biopolymers, allowing the achievement of specific and targeted biological functions. Electrofabrication, which uses a convenient electrical signal input and a mild, modifiable aqueous reaction system, offers a simple, rapid, and highly customizable technique for creating novel materials. Importantly, this approach presents significant promise for expanding the bioactive functions of conventional basic materials, such as collagen, thereby opening new avenues for advanced material design in various biomedical applications.

## 2 Results and Discussion

### 2.1 Salt Effect on Electro‐Assembly of Collagen and Pore Generation

**Figure 1:**
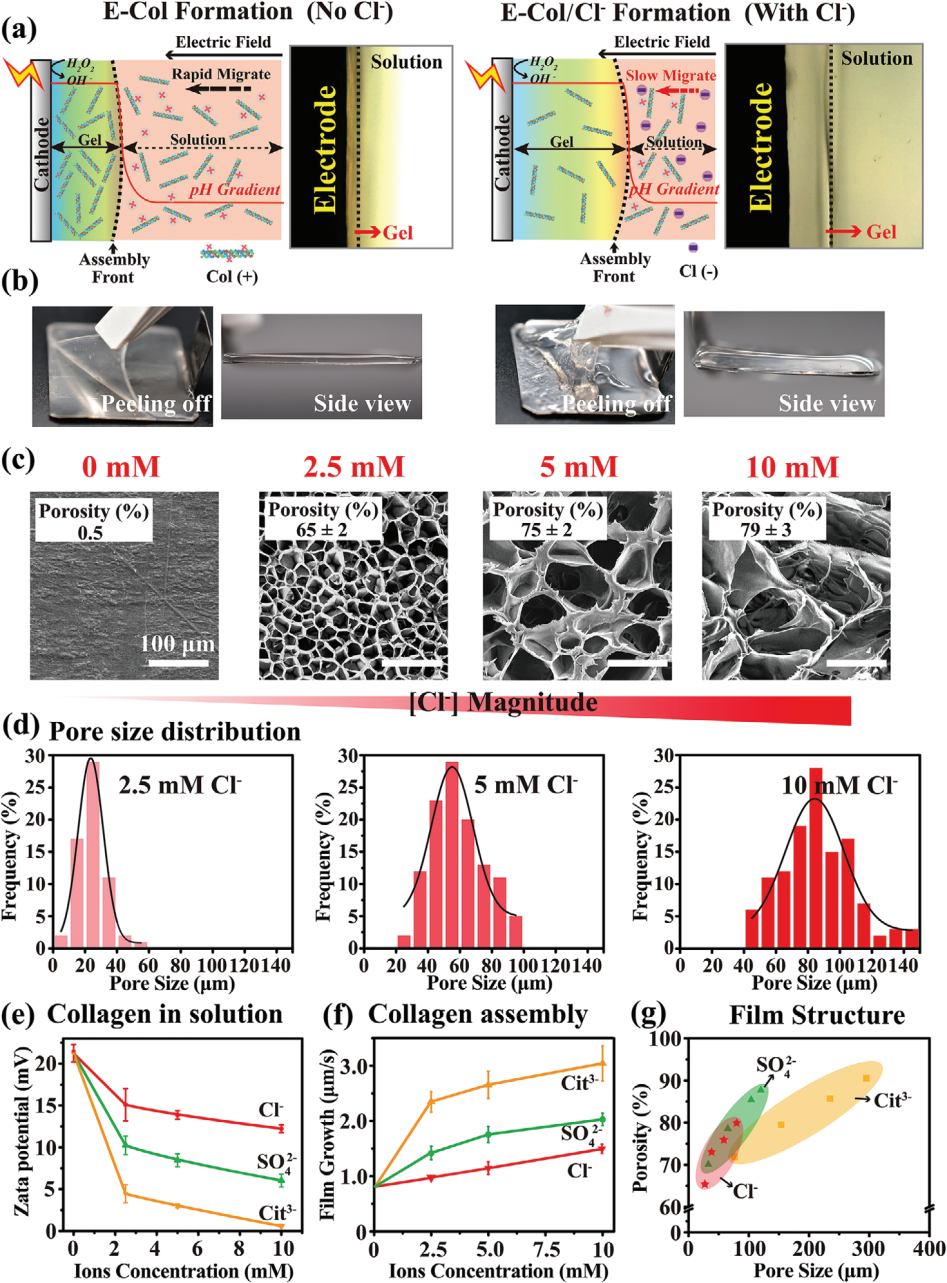
Salt effect on electro‐assembly of collagen and pore generation. a) Illustration of the electro‐assembly of collagen in the absence and presence of soluble NaCl. b) The collagen film (designated as “E‐Col”) electro‐assembled under an environment absence of salts is thin and robust, while the film (designated as “E‐Col/Cl^−^”) electro‐assembled under an environment presence of sodium chloride is thicker and softer (note: electro‐assembly conditions is 6.67 mA/cm^2^ for 1000 s). c) SEM images show that the porosity of the film increases with the concentration of soluble Cl^−^ salt in the electrolyte. d) Surface pore size distribution histograms of films prepared with different concentrations of Cl^−^. (*n* = 100). e) The zeta potential of dissolved collagen molecules under various environments with different concentrations of Cl^−^, SO_4_^2−^, and Cit^3−^ salt species, indicating the charge of collagen molecular gradually decreases with increasing soluble salt concentration. (*n* = 3). f) Quantitative determination of collagen film growth rate in the presence of varying amounts of different salts. (*n* = 5). g) The addition of salts with high anion valences allows a wider range of tuning of pore size and porosity of the electro‐assembled collagen films after freeze drying.

Acid‐solubilized collagen I from porcine skin is dissolved in acetic acid solution to form a transparent collagen molecular solution (designated as “Col”; pH 3.5; Ip 4.5) (zeta potential and circular dichroism [CD] spectrum in Figure S1 (Supporting Information) show right‐handed triple helical collagen I). **Figure** **1**a illustrates the electroassembly of collagen in the absence and presence of NaCl. Mechanistically, such Col solutions (H_2_O_2_ added as sacrificial reductant for OH^−^ generation) can be induced to self‐assemble onto electrodes to form hydrogel films by imposing a cathodic voltage when the solution pH is elevated to the collagen's Ip 4.5. The addition of NaCl to the Col solution screens the imposed electric field such that collagen molecules in the presence of high salt concentrations show little field‐induced molecular migration. This salt effect on the migration of molecules leads to a more rapid growth of the gel front on the electrode surface (for a given current density) and the formation of hydrogel films with lower collagen content (i.e., lower network density). To demonstrate this, we used a microfluidic device (Figure S2, Supporting Information), which allowed in situ observation of collagen electro‐assembly onto the sidewall of a titanium foil cathode (an adjacent platinum foil was used as the anode). After filling the fluidic channel with the Col solution (1% w/v; pH 3.5; 0.1 m H_2_O_2_) containing 5 mM NaCl or no NaCl addition, the power was set to a constant current density, for instance, 6.67 mA cm^−2^. The video (Movie S1, Supporting Information) shows the kinetic process of collagen electro‐assembly in the absence of NaCl, whereas the left micrograph in Figure 1a shows representative images of collagen assembly on the cathode at 1000 s. For comparison, when we performed electro‐assembly from a Col solution containing 5 mm NaCl under analogous conditions (Movie S2, Supporting Information), the micrograph showed faster gel‐front growth on the electrode surface within the same time.

To further examine the effect of NaCl addition to the Col solution on the resultant hydrogel films, electroassembly of collagen was performed from Col solutions containing varying amounts of NaCl on the electrodes, and the assembled films were measured. Briefly, a Ti foil (2.5 cm × 2.5 cm) working electrode was immersed into a Col solution (1% collagen; pH 3.5) containing varying amounts of NaCl (range from 0 to 10 mm) and H_2_O_2_ (0.1 m) and a cathodic potential was applied (6.67 mA cm^−2^ for 1000 s). The photographs in Figure 1b show the electro‐assembled collagen film prepared in the absence of NaCl (designated as “E‐Col”) are thin and robust while the films prepared in the presence of the NaCl (5 mm; designated as “E‐Col/Cl^−^”) were thicker and softer. After deposition, both films were readily peeled from the cathode surface.

The electro‐assembled collagen films were freeze‐dried and their microstructures were investigated using SEM. The surface morphologies of the films are shown in Figure 1c. Films assembled in the absence of NaCl (E‐Col) had a compact structure, whereas films assembled with NaCl (E‐Col/Cl^−^) were porous with interconnected pores. Importantly, the SEM images show that the pore size of these films increased with increasing concentrations of the Cl^−^ salt in the electrolyte. Quantitative analysis of the pore size distribution was performed using ImageJ Plus software. Figure 1d shows the pore size was increased from 20 to 80 µm with increasing of the soluble Cl^−^ concentrations. In addition, we measured the porosity of these films using a gravimetric method, the porosity of these films increased from ≈65% to 79%, and the modulation of porosity and pore size were linked; larger pore size generally resulted in a larger porosity value.

In addition, two other sodium salts with higher anionic valences (sodium sulfate and sodium citrate) were used as salt‐effect agents. The results showed that the addition of these two salts also induced pore formation in assembled collagen films after freeze‐drying, and also showed the ability of ionic concentration‐dependent tuning of internal microstructures (i.e., pore size and porosity) (SEM images and pore size distribution are shown in Figure S3, Supporting Information). Interestingly, tuning of the pore size and porosity within the assembled collagen films seems to be influenced by the valence state of the anions of the added salts. The films assembled using the salt species with a higher anionic valence state demonstrated a larger pore size and porosity under identical ionic concentration conditions, and the relationship between the internal pore size and porosity within the films assembled using the three salt species was E‐Col/Cit^3−^ > E‐Col/SO_4_^2−^ > E‐Col/Cl^−^. This is potentially because the higher the valence state of anions at the same concentration, the stronger the shielding effect on the electric field.

Salt addition can affect the charged state of individual collagen molecules in solution, potentially affecting their response to an electrical field. To better understand how salts modulate the electroassembly of collagen, we systematically investigated the effects of different salt species. Figure 1e shows that the zeta potential of the dissolved collagen molecules (0.02% Col; pH 3.5) progressively decrease in the presence of increasing concentrations of the soluble salts. At the same molar concentration of salt, the solutions with added salt species in a higher anionic valence state demonstrated a more dramatic decrease in the zeta potential of the collagen molecules. The addition of salts further affected the kinetics of the collagen assembly and the resultant films. Figure 1f shows the quantitative results of the collagen film growth rate in the presence of varying amounts of different salt species, and the water content of the resultant assembled films (shown in Figure S4, Supporting Information). These results indicate that the addition of salt to the Col solution results in the rapid growth of polymer films with a higher water content (i.e., lower network density); additional details of the rheological measurements are shown in Figure S4c, Supporting Information), and these effects on the assembly process and the resultant film are positively related to the salt concentration and valence state of the anions. Figure 1g summarizes the range of pore size and porosity in the assembled collagen film tuned by different salt species within the observed salt concentration range (i.e., 2.5 mM to 10 mM). In summary, while the salt effect on electro‐assembly of collagen is an extremely complex process, these observations in Figure 1 illustrate the rich design scope for customizing the internal microstructure (i.e., the porous structure) of the collagen film, potentially as utilizable “physical signals” for periodontitis treatments application.

### 2.2 Electrofabrication of Porous Collagen Film with Bioactive Salt and Salt Delivery by the Film

**Figure 2:**
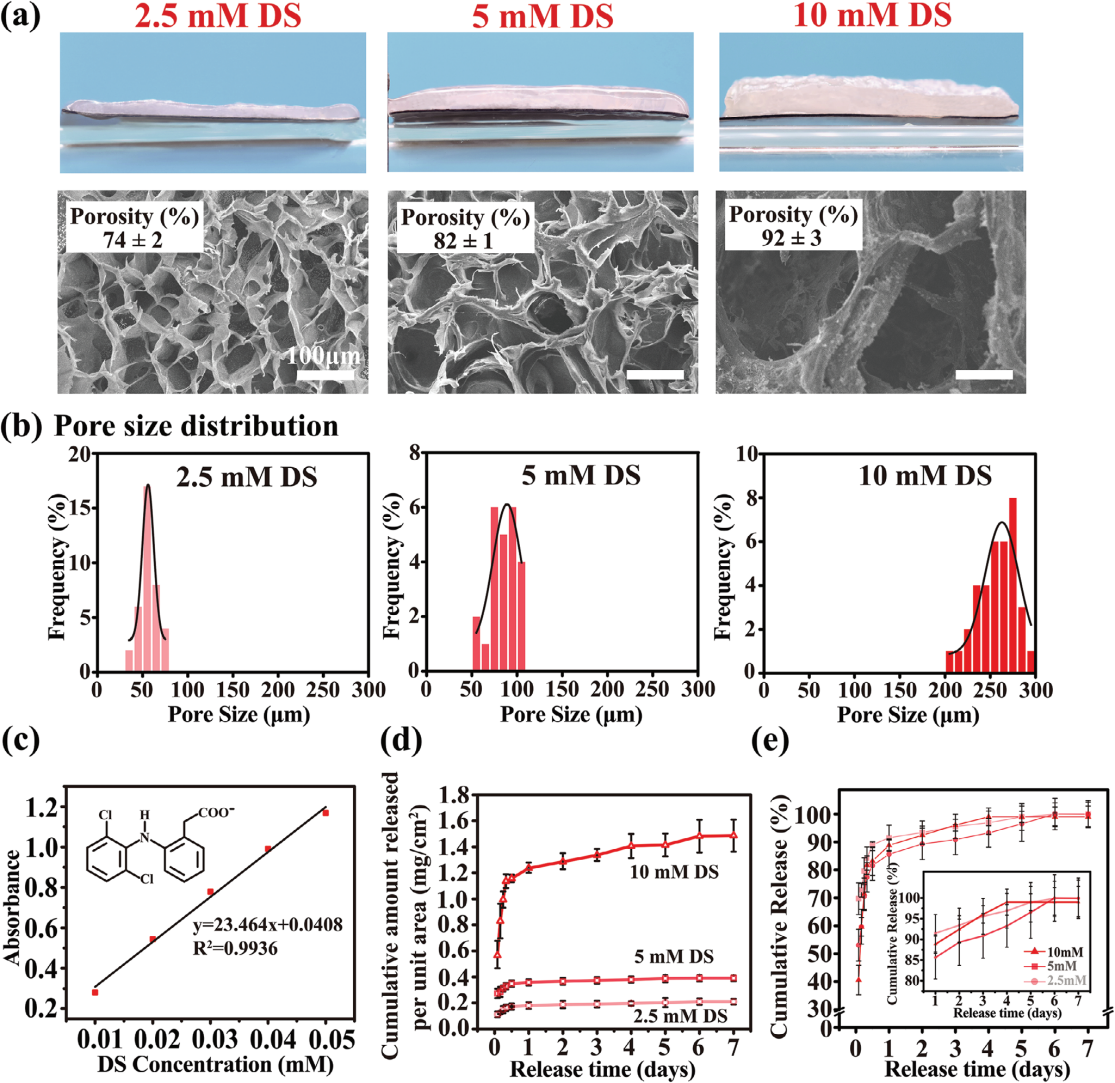
The electrofabrication of porous collagen films using DS. a) Optical cross‐sectional images of the collagen films fabricated using varying concentration of DS (note: electro‐assembly conditions is 6.67 mA cm^−2^ for 1000 s), indicating the thickness of hydrogel films (designated as “E‐Col/DS”) is increasing with the concentration of DS. b) The SEM images of the surfaces of E‐Col/DS hydrogel films after freeze drying, revealing that the film's porosity and surface porous size are increasing with the increasing concentration of DS. b) Histogram of pore size distribution on the surface of E‐Col/DS film prepared with different concentrations of DS. c) Standard calibration curve for DS. The release kinetics of d) cumulative amount curves and e) cumulative released percentage per unit area of E‐Col/DS films fabricated using varying concentrations of DS within 7 days. (*n* = 4).

In addition to the common soluble salts mentioned above, certain biologically active salts, such as inorganic calcium phosphate salts (i.e., hydroxyapatite and tricalcium phosphate) that can induce bone regeneration, sodium salicylate salt used as an anti‐inflammatory and analgesic drug, and disodium pamidronate salt employed in the clinical treatment of osteoporosis, can also induce pore generation in electroassembled collagen films (Figure S5, Supporting Information). DS, a class of nonsteroidal anti‐inflammatory drugs, demonstrate the ability to inhibit the production of inflammation‐associated mediators (e.g., cyclooxygenase). DS is an effective drug for reducing the inflammatory response, making it a promising treatment option for conditions such as periodontitis.^[^ ^17^ ^]^ DS can completely dissolve within a designated concentration range, leading to dissociation of the corresponding anions (i.e., diclofenac ions) (see Figure S6, Supporting Information). We first evaluated the ability of varying DS concentrations to induce pore formation in the electroassembled films. The assembled DS/collagen hydrogel film had a milky white and translucent appearance. This may be due to separation of the polymer and aqueous phases caused by electrostatic interactions between the collagen molecules and diclofenac ions within the film. The optical photograph in **Figure** **2**a shows the thickness of the assembled gel films under the same assembled conditions (6.67 mA cm^−2^; 1000 s; the films are designated as “E‐Col/DS”), which increases with the DS concentrations in electrolytes. Further examination of the SEM images revealed that freeze‐dried E‐Col/DS films exhibited concentration‐dependent changes in their internal porous structure. Generally, higher DS concentrations in the electrolyte result in higher porosity and a larger pore size distribution in the prepared films, as shown in Figure 2b. These findings are consistent with the observations shown in Figure 1, suggesting that the inclusion of DS in the electrolyte induces the formation of porous structures within the freeze‐dried collagen films and allows for concentration‐dependent customization of the porous structure (the morphology of the freeze‐dried films fabricated using varying current densities is displayed in Figure S6, Supporting Information).

Notably, the assembled E‐Col/DS film incorporated a portion of DS added to the electrolyte, allowing for its subsequent release from the freeze‐dried porous film. The release of DS from the freeze‐dried films in PBS was further monitored using a UV spectrophotometer (the drug‐loading rate and loading capacity are shown in Figure S6, Supporting Information). DS exhibits a maximum absorbance at λ max = 276 nm, and Figure 2c presents the standard equation relating absorbance (y) to concentration (x, mg/mL) as y = 23.464x + 0.0408 (with a correlation coefficient of R2 = 0.9936 and within a linear range of 0.01–0.05 mg mL^−1^), enabling the quantification of DS released into PBS. Figure 2d shows the cumulative amount released per unit area of E‐Col/DS films prepared with varying DS concentrations. The results indicate that the E‐Col/DS films fabricated using electrolytes with higher DS concentrations exhibited higher DS release, presumably because of the higher initial DS content in the films. Moreover, Figure 2e illustrates the cumulative release percentage of DS from the E‐Col/DS films, showing a rapid release of DS on day 1, with the remaining DS in the film completely released within 7 days. It should be noted that fully immersing the film in solution represents an extreme condition for measuring the release kinetics of DS, and DS can directly dissolve from E‐Col/DS into water, facilitating rapid release. However, the limited amount of liquid in the in vivo environment means that the release period of DS into the body can be prolonged. It has been speculated that the release of DS into the body is largely influenced by film degradation.

### 2.3 Electrofabrication of Collagen/DS Janus Composite Films

**Figure 3:**
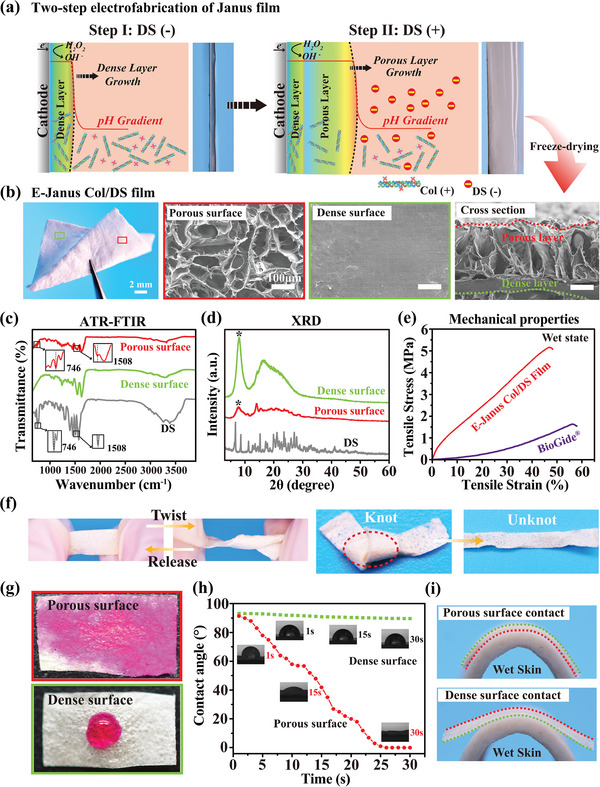
Electrofabrication of Janus porous collagen/DS films and its characteristics. a) Illustration of electrofabrication of Janus films (designated as “E‐Janus Col/DS”) using a two electro‐assembly steps method. b) Optical image of the freeze‐dried E‐Janus Col/DS film and its SEM images show the macroscopic and microscopic morphological details of E‐Janus Col/DS films. One surface is dense and without pores, while the other surface is loose and porous. Besides, the crosssection of Janus film demonstrates the dramatic transition from the porous layer to the dense layer [note: the up‐interface (i.e., porous face side) and low‐interface (i.e., dense face side) of the Janus film are marked by red and green dashed lines respectively]. c) ATR‐FTIR and d) XRD spectra further respectively proves the asymmetric chemical and crystalline structures of Janus film. e) Representative stress–strain curve of wet E‐Janus Col/DS film, indicating its higher tensile stress than the commercial Bio‐Gide film. f) Optical images show the Janus film can be twisted or knotted and allowed to return to its original shape. g) Photographs taken after liquid drops were contacted with the dense surface or porous surface of the Janus film. h) Dynamic water contact angle measurements for the two surfaces of the Janus Col/DS film demonstrate that the porous face promotes liquid spreading and exhibits higher hydrophilicity compared to the dense face. i) The optical images of the dense face and porous face of the E‐Janus Col/DS film adhere to the surface of wet pigskin.

Next, we fabricated DS contained Janus collagen films by sequential electro‐assembly of collagen in a pre‐programmable salt environment, as shown in **Figure** **3**a. Experimentally, we prepared our Janus collagen/DS composite films (designated as “E‐Janus Col/DS”) in two steps: In the first step, the dense collagen layer was fabricated by assembly from a collagen solution (collagen 1%; pH 3.5; 6.67 mA cm^−2^; 500 s). In the second step the porous composite layer was assembled from a collagen solution (collagen 1%) containing DS (5 mm) using 6.67 mA cm^−2^ for 1000 s. It should be note that, the thickness of the dense and porous layers (fabricated under specific salt concentration and current density) in the Janus film can be adjusted by varying the assembly time at different steps, as we have previously reported.^[^ ^15^, ^16^ ^]^ The photo in Figure 3a further shows the milky and opaque appearance of the collagen/DS composite gel fabricated by two‐step electro‐assembly. The optical images shown on the left of Figure 3b show that the E‐Janus Col/DS film obtained by freeze‐drying of the gel has flexible mechanical properties, the SEM image on the right shows that the two surfaces of the E‐Janus Col/DS film have a dense and porous morphology, and the cross‐sectional SEM image shows that the porous and dense layers are closely combined. Even after the tensile fracture, the dense and porous layers of the Janus film remained relatively tight and did not separate, as shown in Figure S7, Supporting Information). Presumably, the stable binding between the dense and porous layers comes from hydrogen bonding between the collagen molecules and the covalent bonding generated by subsequent cross‐linking. The collagenous BioGide film (Geistlich Pharma, Wolhusen, Switzerland) is one of the most widely used commercial GBR films, while it shows relatively fewer internal porous space (the surface and cross‐sectional morphology of BioGide film is shown in Figure S8, Supporting Information), presumably the BioGide film potentially allows adhesion and proliferation of cells only on the surface. Chemical analysis confirmed that the two faces of the electrofabricated E‐Janus Col/DS film have different chemical compositions, and the ATR‐FTIR spectra in Figure 3c show peak signatures for DS in the porous layer but are absent from the dense layer (note: the EDS mappings of the film's porous layer are shown in Figure S9, Supporting Information). The XRD spectra in Figure 3d further indicates the different crystalline structures of the dense and porous surface of E‐Janus Col/DS film, and compared with the dense surface of the E‐Janus Col/DS film, the porous surface of E‐Janus Col/DS film shows a significantly weakened crystallization peak.

Figure 3e shows the representative stress–strain curves of the wet E‐Janus Col/DS film and BioGide film, as can be seen, E‐Janus Col/DS films exhibit higher strength than BioGide films, presumably it mainly due to the high packing density of the dense layers in E‐Janus Col/DS films (1.05 g cm^−3^, and the packing density of BioGide film is 0.3 g cm^−3^), and the summarized tensile strength and strain at failure at wet state and the mechanical property measurements for dried films are shown in Figure S10 (Supporting Information). The images in Figure 3f illustrate the flexibility of the E‐Janus Col/DS film on a 10 cm ×10 cm Ti foil cut into rectangular shapes. The first image shows that the wet film (1×3 cm) can be twisted by 360° and upon release returns to its original state. The second image shows that a dry film (0.5×6 cm) can be tied into a knot and untied without tearing. These images illustrate the relative ease of handling the E‐Janus Col/DS films. Although Janus films with larger pore sizes and higher levels of DS release fabricated with high DS concentrations may provide greater immunomodulatory activity (i.e., reduced inflammation),^[^ ^17^, ^18^ ^]^ they exhibit poor structural stability under dynamic mechanical environments. The E‐Janus Col/DS films with a porous layer fabricated with 5 mm DS were chosen as the preferred group for the follow‐up experiment because of their stable mechanics and the absence of fracture, mechanical degradation, and significant irreversible deformation during cyclic loading, as shown in Figure S11 (Supporting Information).

The microstructural differences between the two faces of the Janus film resulted in differences in their properties, as illustrated by wettability measurements. In this study, water droplets (20 µL, containing 1 mm Rhodamine) were placed on the different surfaces of E‐Janus Col/DS film. As illustrated in the photographs in Figure 3g, drops placed on the porous surface were completely absorbed, whereas those placed on the dense surface were retained. A more quantitative analysis is illustrated in Figure 3h which shows a rapid decrease in the contact angle for a drop placed on the porous surface, whereas the contact angle for the dense surface remains relatively constant over the 30 s measurement time. [Note: the porous faces of Janus Col/DS film fabricated with various DS concentrations (i.e., 2.5 to 10 mm) present different degrees of hydrophilicity (Figure S12, Supporting Information)]. Although the Janus porous film exhibited good wettability, it also demonstrated appropriate water absorption without excessive swelling in moist environments (Figure S13, Supporting Information). Another consequence of the property differences between the two faces of the E‐Janus Col/DS film is that they exhibit different adhesion properties when in contact with wet pigskin surfaces, as shown in Figure 3i.

### 2.4 Anisotropic Cell Guidance by E‐Janus Col/DS Film

**Figure 4:**
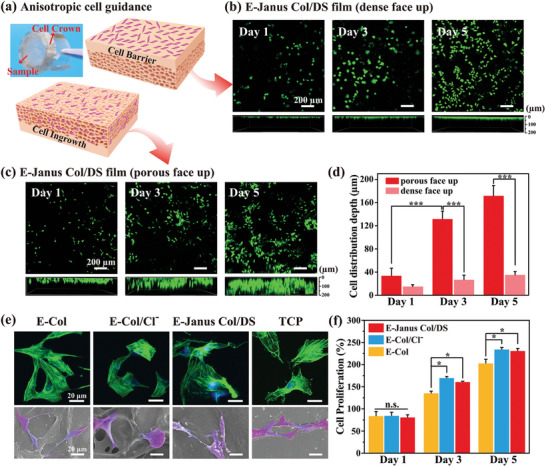
The Janus porous structure of the E‐Janus Col/DS film provides anisotropic guidance for fibroblasts and its porous surface facilitates the adhesion and proliferation of osteoblasts. a) The experimental approach involves a cell crown insert sealed with a film (either with the dense face up or the porous face up), positioned on a well plate to isolate upper and lower volumes for assessing anisotropic cell guidance. b–d) 3D fluorescence images demonstrate that when L929 fibroblast cells were seeded onto the Janus Col/DS film, they did not penetrate the dense layer but instead grew into the porous layer (*n* = 5). e) Confocal and SEM images of MC3T3‐E1 osteogenic precursor cells (marked in pseudo‐purple color) depict cell adhesion in the porous layer of the Janus Col/DS film. f) Comparison of the proliferation of osteogenic precursor cells (MC3T3‐E1) on different films suggests that the Janus porous structure promotes cell proliferation (*n* = 4). ^*^ *p* < 0.05, ^***^ *p* <0.001. All data are presented as mean ± SD. One‐way ANOVA was used for comparison.

We then conducted experiments using L929 fibroblasts to evaluate cell growth on the two distinct surfaces of the E‐Janus Col/DS film (6.67 mA/cm^2^, 1000 s, dense layer with ≈50 µm in depth, porous layer with ≈250 µm and 82% porosity), with the aim to determine how surface topography influence cell movement. These experiments utilized the setup depicted in **Figure** **4**a, where Janus Col/DS films were used to seal the cell crown inserts (with either the dense side facing up or the porous side facing up) and were placed on well plates to separate the two volumes. Fibroblasts were then introduced into the upper volume and their growth and distribution within the Janus Col/DS film were evaluated after various incubation periods. For analysis, live/dead staining of cells at different time points was performed, and cell viability was assessed using confocal laser microscopy (green staining). Figure 4b shows the fluorescence images illustrating the growth and distribution of fibroblasts seeded on a dense surface. The top images represent the film surface, whereas the bottom images display the optical cross‐section. These images consistently show an increase in the number of live cells, indicating that these fibroblasts proliferated throughout the 7‐day incubation period. Fluorescent cross‐sectional images revealed that the cells were primarily localized within a narrow region adjacent to the dense surface layer of the Janus Col/DS film throughout the culture process. In contrast, Figure 4c shows that when the fibroblasts were seeded on a porous surface, not only do they proliferated and infiltrated the porous structure.

To compare the depth of cell penetration on the different surfaces of the Janus Col/DS film, we analyzed the cross‐sectional fluorescent images (note that because of the film's unevenness, accurately determining the film surface for a precise depth analysis was challenging; thus, the analysis of the penetration depth was semi‐quantitative). Figure 4d shows that the fibroblasts exposed to the dense surface remained confined to a narrow region near the surface throughout the 7‐day culture period. Conversely, when fibroblasts were exposed to a porous surface, they penetrated deeper into the interior regions of the film. These findings demonstrate that the dense layer of the Janus Col/DS film serves as a barrier to prevent cell penetration, potentially impeding the invasion of fibrous connective tissue into AB defects.

To evaluate the significance of the porous structure on cell growth, we compared the ability of various films to support osteoblast proliferation. These films included: i) the “Janus Col/DS” film with a porous layer porosity of 82%; ii) a nonporous collagen film prepared by electro‐assembling collagen without soluble salts addition (i.e., E‐Col film), as described in Figure 1; iii) a porous collagen film prepared by electro‐assembling collagen using electrolyte with presence of soluble NaCl (i.e., E‐Col/Cl^−^), with a similar porosity (≈82%) to that of the Janus Col/DS film. We seeded the osteoblast precursor cell line MC3T3‐E1 on the porous surface of Janus Col/DS and Porous E‐Col/Cl^−^ films, as well as on the non‐porous surface of the E‐Col film. The confocal fluorescence and SEM images in Figure 4e demonstrate that after just one day of seeding, osteoblast cells adhered to the porous surfaces of both films, as well as the non‐porous surface of the E‐Col film. Both the fluorescence and SEM results (marked by a pseudo‐purple color) revealed excellent cell spreading, characterized by filamentous pseudopodia and unidirectional lamellipodia.

Furthermore, we quantitatively analyzed the proliferation of MC3T3‐E1 cells on the different film surfaces. Experiments were conducted by seeding 5 × 10^4^ osteoblasts per well on different film surfaces, culturing them for varying durations, and measuring their proliferation using a Cell Counting Kit‐8 (CCK‐8, Dojindo). Figure 4f illustrates a comparison of osteoblast proliferation on these different films at various time points [note: 100% was normalized to the proliferation observed on a tissue culture polystyrene plate (TCP) on the first day of measurement]. We observed that all films supported cell proliferation, although the proliferation of all films after one day of incubation was slightly lower than that observed on TCP. Importantly, Figure 4f shows significantly higher osteoblast proliferation on the two porous surfaces of the Janus Col/DS and Porous E‐Col/Cl^−^ films than on the non‐porous surface of the E‐Col film.

These observations are consistent with the expectation that cell proliferation would be enhanced by cultivation in three dimensions (vs cultivation on 2D surfaces)^[^ ^19^ ^]^ and that the porous structure presumably promotes proliferation by providing more space for cell adhesion and rapid nutrient transport, which have the potential to promote osteogenesis when contacting osteogenic cells.

### 2.5 In Vitro Anti‐Inflammatory, Osteogenic, and Antibacterial Functions of E‐Janus Col/DS Film

In the context of periodontitis, persistent and severe inflammation of the periodontal tissue leads to initial connective tissue injury and AB resorption, thereby limiting bone regeneration even after treatment. We expect that the porous surface of the E‐Janus Col/DS film when facing the area of bone resorption creates an anti‐inflammatory environment that fosters subsequent osteogenesis. This is because the porous surface of E‐Janus Col/DS allows the local delivery of DS with anti‐inflammatory activity, as shown in Figure 2. In addition, it has been reported that the porous topology and hydrophilic surface of implants can induce an anti‐inflammatory phenotype in immune cells,^[^ ^20^ ^]^ thus, the hydrophilic porous surface of E‐Janus Col/DS film is potentially another cue to endow the film with an anti‐inflammatory function.

Macrophages play a pivotal role in the regulation of the immune ME in periodontitis and are known for their plasticity in switching between pro‐inflammatory M1 and pro‐healing M2 macrophages. Thus, we investigated the effect of the Janus Col/DS film on the polarization of RAW264.7 (a murine macrophage cell line) using flow cytometry and real‐time quantitative polymerase chain reaction (RT‐qPCR). To evaluate the effect of the porous structure and DS component of E‐Janus Col/DS film for the polarization of RAW264.7, two control films fabricated as above were included as comparison: a porous E‐Col/Cl^−^ film [with a similar porosity (≈82%) to the porous layer of Janus Col/DS film] just can provide a porous topology and without DS local environment; and a nonporous E‐Col film can provide a non‐porous topology and no DS local environment as a negative control group; and TCP was set as blank group.

**Figure 5:**
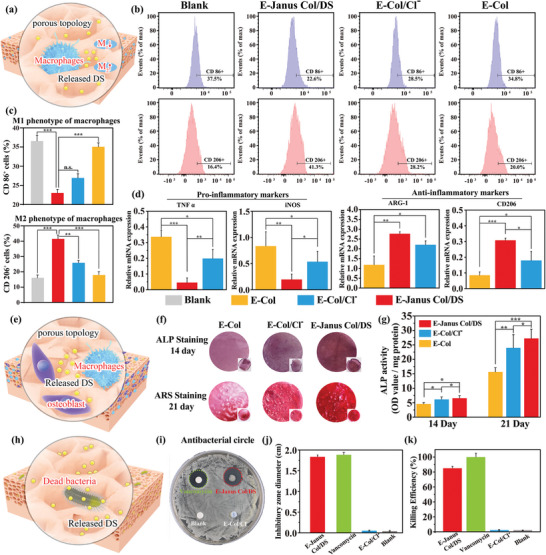
In Vitro anti‐inflammatory, osteogenic, and anti‐bacterial functions of E‐Janus Col/DS film. a) Illustration of the LPS activated macrophages (i.e., RAW264.7) cultured on the DS‐loaded porous surface of E‐Janus Col/DS film. b) Representative flow cytometry results of CD 86**+** (M1 markers) and CD206**+** (M2 markers) expression of macrophages incubated on TCP (i.e., blank group), the non‐porous surfaces of E‐Col films, the porous surfaces of E‐Col/Cl^−^ and E‐Janus Col/DS films for 1 day and c) the quantitative percentages of M1 and M2 polarization of RAW 264.7 in different groups (*n* = 4). d) The pro‐inflammatory and anti‐inflammatory related genes expression of RAW264.7 after 24 h culturing on the films surface using qPCR, which indicating the loaded DS and porous surface structure of E‐Janus Col/DS film are both important for decreasing the inflammatory (*n* = 4). e) Illustration of RAW264.7 and MC3T3‐E1 cells co‐cultured on the porous surface of the film. f) ALP and Alizarin Red S staining of cells seeded on the surface of different films’ surfaces for 14 days and 21 days, respectively, and g) the quantitative ALP activity results indicating that the MC3T3‐E1 cells present increasing osteogenic differentiation on the porous surface of E‐Janus Col/DS film (*n* = 4). h) Antibacterial activity of E‐Janus Col/DS film against Staphylococcus aureus. i) Inhibition zone experiment of different samples. Quantification of j) inhibition zone diameter and k) killing efficiency rate (*n* = 5). ^*^ *p* < 0.05, ^**^ *p* < 0.01, ^***^ *p* <0.001. All data are presented as mean ± SD. One‐way ANOVA was used for comparison.

RAW 264.7 cells were co‐cultured with different groups of films for 24 h, as illustrated in **Figure** **5**a. (Note: Figure S15, Supporting Information shows the adhesion of macrophages to the different groups of films). Subsequently, the cells were then stained with FITC rat anti‐mouse CD86 and APC rat anti‐mouse CD206 to determine the M1 and M2 phenotypes, respectively, by flow cytometry. The representative flow cytometry results and the quantitative percentages of M1 and M2 polarization of RAW 264.7, are shown in Figure 5b,c. The results showed that compared with the blank group, the co‐culture groups of RAW 264.7 cells seeded on different films’ surfaces showed lower levels of M1 phenotype (22.6%, 28.5%, and 34.8% in E‐Janus Col/DS, E‐Col/Cl‐ and E‐Col, respectively). The cells seeded on the porous E‐Janus Col/Cl‐ film showed higher polarization of the M2 phenotype and less polarization of M1 phenotype than those seeded on the dense E‐Col film, indicating that the porous morphology is indeed beneficial for the transformation of macrophages from M1 towards M2 phenotype. Notably, the E‐Janus Col/DS presented the highest polarization of M2 phenotype, which could be ascribed to the fact that E‐Janus Col/DS not only provided a porous surface, but also continuously released the active drug DS with anti‐inflammatory properties.

For the qPCR assay, lipopolysaccharide‐activated RAW264.7 activated by cells were seeded onto the surfaces of different films, and then cells were lysed after 24 h of culturing to detect the expression of the M1 and M2 phenotype‐related genes. The gene expression level of each group was normalized to the tissue culture polystyrene plate (TCP) group, Figure 5d shows that RAW264.7 cultured on the porous surface of E‐Janus Col/DS films expressed the lowest levels of M1 phenotype markers (TNF‐α and iNOS) and the highest levels of M2 phenotype markers (ARG‐1and CD206) compared to those cells cultured on the surfaces of dense and porous collagen films without DS containing, presumably the rapidly released DS from E‐Janus Col/DS films enable directly polarize the macrophages to M2 phenotype and inhibit inflammation. Interestingly, the porous E‐Col/Cl^−^ film control showed the lower levels of M1 phenotype markers expression and the higher levels of M2 phenotype markers expression compared to the nonporous E‐Col film control, and this observation suggests the porous topology benefits to the M2 phenotype of macrophages. In general, these results revealed that the E‐Janus Col/DS film enable promote the macrophages polarize to M2 phenotypes by coupling the local delivery of “molecular” cues (i.e., DS) and the “physical” cues (i.e., film's porous topology).

The ability of the Janus Col/DS film to induce osteogenic differentiation in the context of the immune environment was further evaluated in vitro using the osteoblast precursor cell line MC3T3‐E1 and LPS‐activated macrophages. Experimentally, LPS activated RAW264.7 was mixed with MC3T3‐E1 cells (with a density of 5 × 10^6^ cells) at a ratio of 4:1, and then directly co‐cultured on the surface of above various films as illustrate in Figure 5e, and after varying times osteogenic markers were measured. One marker of osteogenic differentiation is alkaline phosphatase (ALP). Images of the ALP‐stained films after 14 d of co‐culture are shown in the top panel of Figure 5f. Visually, the porous E‐Col/Cl^−^ film showed a darker ALP staining compared to the nonporous E‐Col film, indicating beneficial porous topology of film for osteoblast differentiation in the presence of macrophages. This may be due to M2 phenotype macrophages induced by the porous structure promoting the osteogenic differentiation of MC3T3‐E1 cells through paracrine action. We then found more significant ALP staining on the porous E‐Janus Col/DS film, which was presumably due to the release of DS, which further intervenes in the cell phenotype.

The second marker was alizarin red S staining (ARS), which measures calcium mineral formation. ARS staining was performed on the films after 21 d of incubation, and the results are shown in the bottom panel of Figure 5f. Darker ARS staining (i.e., greater mineral nodule density on the material surface) was observed in the E‐Janus Col/DS film than in the E‐Col/Cl^−^ and E‐Col films. Quantitative analysis of ALP activity was performed on days 14 and 21. The results shown in Figure 5g indicate that MC3T3‐E1 cells underwent different degrees of osteogenic differentiation in all groups. At both time points, the porous surfaces of the E‐Janus Col/DS film and the porous surface E‐Col/Cl^−^ film without DS remarkably promoted osteogenic differentiation compared to the non‐porous surface, while no statistical difference was observed between E‐Janus Col/DS and E‐Col/Cl^−^. This observation further demonstrates the importance of porous topology in osteogenesis in the context of the immune environment.

Another potential function of the E‐Janus Col/DS film is its antibacterial activity, since DS released from the porous layer is reported to inhibit the DNA synthesis of bacteria^[^ ^21^ ^]^ as illustrated in Figure 5h. Staphylococcus aureus, a common foodborne pathogenic microorganism that causes periodontitis, was used to evaluate the antibacterial properties of E‐Janus Col/DS films. In addition, various other control films were used for comparison with the E‐Janus Col/DS films. These films include: i) a filter paper loaded with vancomycin (5 mg mL^−1^ an antibiotic against *S. aureus*); ii) a filter paper without loaded vancomycin as a blank; and iii) a porous film with a similar porosity to the porous layer of the E‐Janus Col/DS film (note: it is fabricated using an electrolyte containing sodium chloride), and it is denoted as E‐Col/Cl^−^ film. The antibacterial activity of the E‐Janus Col/DS film was further demonstrated by inhibition zone assays with *S. aureus*. In these experiments, a film sample was placed on the surface of an agar plate after inoculation with bacteria. After incubation for 24 h, Figure 5i shows the growth inhibition zone in both the E‐Janus Col/DS (marked with red circles) film and positive control group loaded with vancomycin filter paper (marked with blue circles). For the two control films (filter paper without loaded vancomycin and E‐Col/Cl^−^ film), no inhibition zone was observed. Figure 5j further quantifies the diameter of the inhibition zone, and the results show that the E‐Janus Col/DS film had an inhibition zone diameter comparable of the inhibition zone compared with filter paper loaded with vancomycin. In addition, various films were co‐cultured with *S. aureus* (10^6^ CFU/mL) for 18 h, and the survivors were counted using dilution plate counting (Figure S16, Supporting Information).^[^ ^22^ ^]^ Killing efficiency was calculated by comparing the bacterial colonies formed when *S. aureus* was cultivated in the presence of films and in growth media without films. Figure 5k shows the comparable kill efficiency of E‐Janus Col/DS film (85%) was slightly lower than that of loaded with vancomycin filter paper (98%). In general, these results prove that the E‐Janus Col/DS film has an anti‐bacterial function and potentially provides beneficial properties for the treatment of periodontitis.

### 2.6 In Vivo Tissue Responses and Degradation of E‐Janus Col/DS Film

We next evaluated the effect of Janus porous structure and DS composition on the in vivo tissue responses and degradation of E‐Janus Col/DS films (dense layer ≈100 µm, porous layer ≈250 µm, and 82% porosity in the porous layer) by implanting them subcutaneously on the left and right sides of the dorsal region of rats. Additionally, we evaluated two control films: non‐porous E‐Col films (≈200 µm) and Janus Collagen porous films without DS prepared by sequential electro‐assembly (it has the analogous structural parameters with above E‐Janus Col/DS film, and NaCl was used for the porous layer formation, designated as “E‐Janus Col/Cl^−^ film”). All the films were partially stabilized by glutaraldehyde crosslinking prior to implantation. Throughout the observation period, all mice remained healthy and no surgery‐related infections or complications were observed. Photographs were taken and dorsal skin tissues with integrated materials were collected during the designated post‐implantation period.

**Figure 6:**
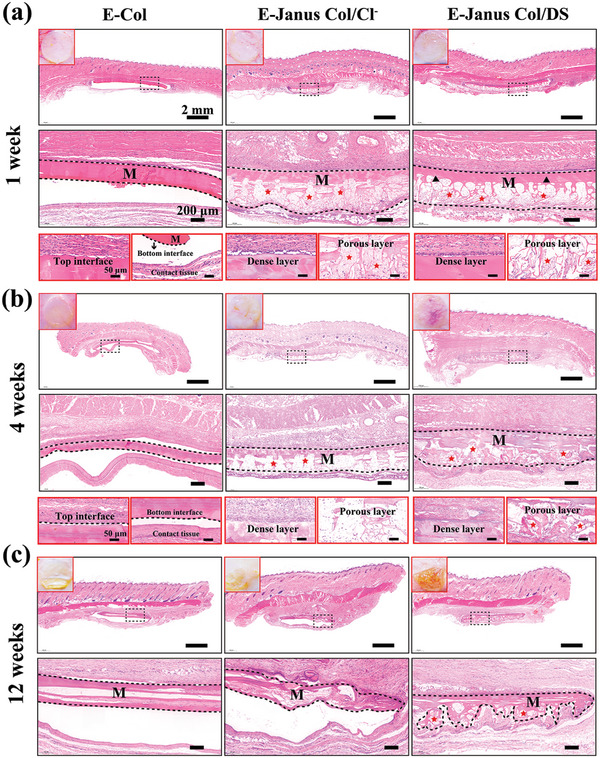
Tissue responses and degradation to different films 1, 4, and 12 weeks after subcutaneous implantation in rats. a) Optical images, H&E staining images with different magnifications for 1 week subcutaneous implantation [Note: black dotted line indicates the area of implanted materials, M; black triangle marks the dense layer of Janus film; red star marks the tissue ingrowth into the Janus porous film]. The H&E staining images with different magnifications for the different film for b) 4 weeks and c) 12 weeks after subcutaneous implantation. (*n* = 4).

The top two rows of **Figure** **6**a show macroscopic photographs (top‐left inset) and histological H&E‐stained tissue sections at low and high magnifications taken 1 week after film implantation. In all cases, the implantation sites appeared healthy, with no noticeable redness or swelling. The low magnification images in the top row of Figure 6a show all the implanted films (designated “M”) remained intact and integrated well with the tissue (i.e., no separation from the upper skin tissue was observed). The higher‐magnification histological H&E‐stained images in the second row reveal that the films from the various groups essentially maintained their structure after 1 week implantation. For both Janus collagen porous film groups, a distinct Janus porous structure was visible (dense layer facing up and porous layer facing down). Furthermore, these images show that both the E‐Janus Col/DS and E‐Janus Col/Cl^−^ films remained intact without significant degradation or damage to the dense layer (marked with black triangles) after 1 week of implantation.

The third row of Figure 6a shows the highest magnification of histological H&E‐stained images and the histological response of the top and bottom surfaces of the different films to tissue contact. The non‐porous E‐Col film groups on the left show that fibroblast cells of the connective tissue could only grow along the film's top or bottom surfaces without penetrating the film's interior, indicating that the non‐porous films can serve as a physical barrier to fibrous tissue penetration. For the two Janus porous films, the images of the dense layers on the left in the third row for the two films show fibrous tissue growth along the surface of the dense layer with no obvious penetration into the film. The images of the porous layers on the right in the third row of the two films show cellular ingrowth (marked with a red asterisk), indicating that the porous structure provides the necessary three‐dimensional space for the inward growth of cells or tissues.

Figure 6b shows macroscopic photographs (top left inset) and histological H&E‐stained tissue sections 4 weeks after implantation under the skin. Macroscopic photographs (top‐left inset) show that after 4 weeks, partial subcutaneous material can still be observed. High‐magnification H&E‐stained images revealed that the films in various groups experienced different degrees of degradation 4 weeks after implantation. For the non‐porous E‐Col films, remnants of the degraded films were still discernible (surrounded by a black dotted line). For the two Janus porous collagen films, the dense layers showed partial degradation but maintained their main structure to maintain their barrier function. The porous layers showed significant degradation and instead grew many new tissues (marked with a red asterisk), further indicating that the porous layer of the Janus film can gradually degrade to provide more space for tissue regeneration. After 12 weeks of subcutaneous implantation of the different films, a large amount of film degradation occurred, and the structure was no longer complete, as shown in Figure 6c.

In addition, the E‐Janus Col/DS film did not exhibit severe inflammation at the initial stage of implantation and showed low expression of inflammation‐related factors (i.e., tumor necrosis factor‐alpha and IL‐6) (Figure S17, Supporting Information).

In summary, the Janus collagen films demonstrated excellent biocompatibility and appropriate degradability in subcutaneous implantation studies. In particular, the Janus Col/DS films presented anisotropic guidance for tissue growth just after 1 week implantation. Within 8 weeks, the porous layer of the Janus films showed partial degradation and was accompanied by the growth of a large number of new tissues, while the dense layer remained relatively intact and maintained its barrier function. The anisotropic microstructure of the Janus Col/DS films appeared to perform both functions, with the dense layer serving as a guide for connective tissue fibroblast growth on the surface and as a barrier to prevent fibrous tissue invasion, whereas the porous layer was conducive to cell and tissue ingrowth. In clinical applications, the performance stability of Janus porous films in the complex environment of the mouth still requires attention (for example the effect of human salivary enzymes on film mechanics, as shown in Figure S18, Supporting Information).

### 2.7 In Vivo Studies of E‐Janus Col/DS Film for Periodontitis Treatment

**Figure 7:**
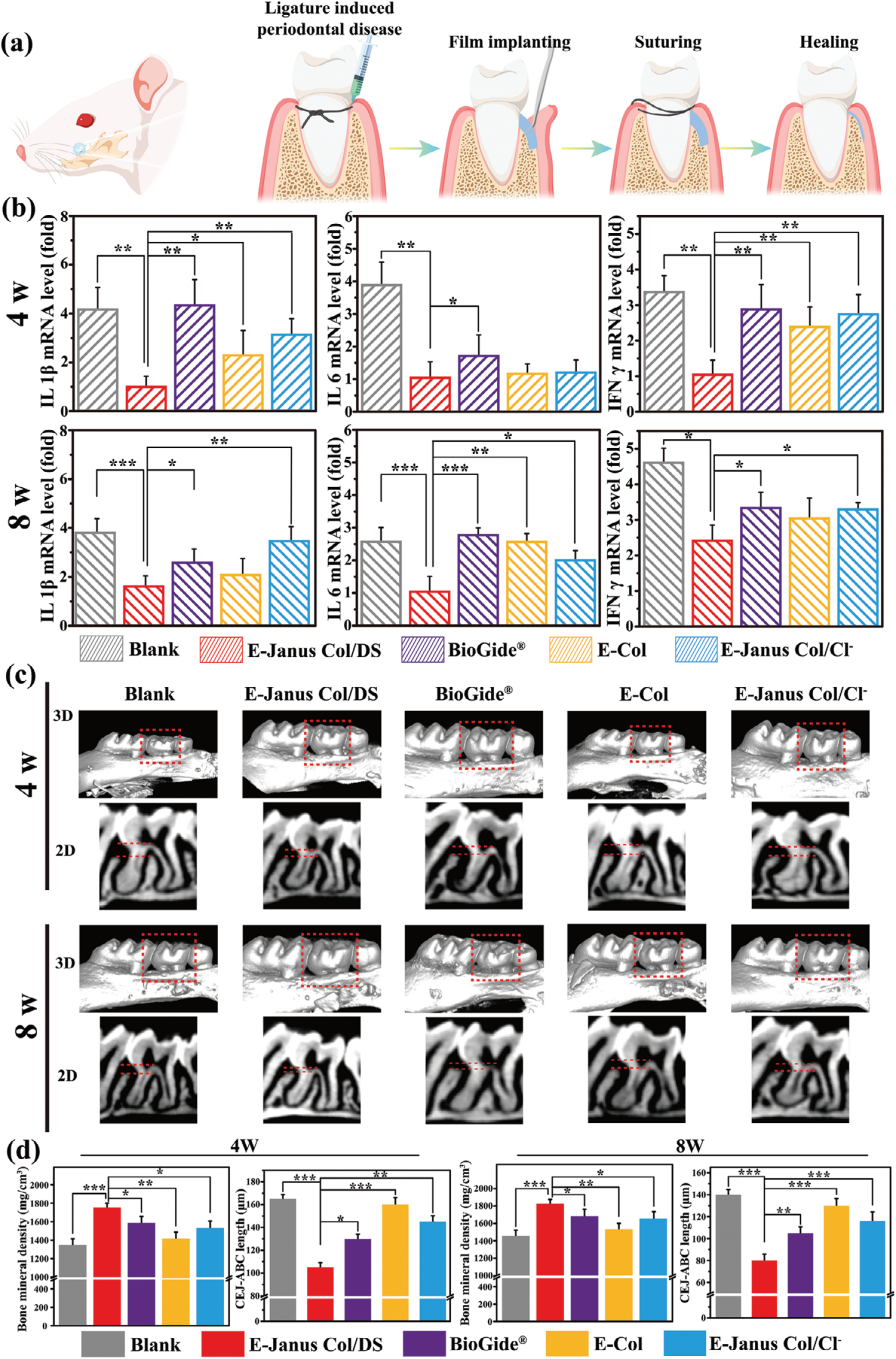
In vivo evaluation of E‐Janus Col/DS film for periodontitis treatment. a) Schematic illustrates the induction of the periodontitis in rats and the procedure of surgery for film implantation. b) Relative mRNA expression levels of the corresponding proinflammatory genes (IL 1β, IL 6, and IFN γ) in the periodontal tissue at 4 and 8 weeks after surgery. c) Micro‐CT images of rat maxillas at 4 and 8 weeks after implantation of different film materials. All specimens were normalized and Micro‐CT images calibrated to enable quantitative comparison, and the distance between the alveolar bone crest (ABC) and the cementum junction (CEJ) marked with the red dashed line. d) Quantification of AB recovery determined by measuring the distance between the ABC and CEJ and the bone mineral density using 3D reconstruction volume after 4 and 8 weeks after surgery. (*n* = 4). ^*^ *p* < 0.05, ^**^ *p* < 0.01, ^***^ *p* <0.001. All data are presented as mean ± SD. One‐way ANOVA was used for comparison.

The functionality of the E‐Janus Col/DS film was tested in vivo using a rat model of periodontitis. In this experiment, the absorption of AB was induced by periodontitis through thread ligation of the maxillary second molar in mice and daily injection of 109 CFU/mL Porphyromonas gingivalis (P.G.) into the ligated thread during the ligation period in 40 rats. After the micro‐CT confirmed AB absorption in the second molar, different films were implanted into the periodontal pocket (**Figure** **7**a). The E‐Janus Col/DS films (with a dense layer ≈100 µm, a porous layer ≈250 µm, and 82% porosity in the porous layer) was chosen as the experimental film, because above in vitro and subcutaneous implantation results showed its long‐term barrier function (associated with the dense layer), as well as its role in reducing inflammation and promoting osteogenesis (associated with the porous layer loaded with DS). Besides, three types of films were included as controls in this study: i) The E‐Col film without a porous layer, which assessed the importance of a porous topology for periodontitis treatment; ii) The E‐Janus Col/Cl‐ film without DS (it has analogous structural parameters to the above E‐Janus Col/DS film, with a porous layer fabricated by NaCl), to evaluate the importance of loaded DS for periodontitis treatment; iii) A commercially available tissue‐derived collagen film, BioGide, which is widely used clinically to guide bone regeneration and allows for comparison with clinical standards. Finally, the inflammation levels and regeneration of periodontal tissue (AB and gingival soft tissue) were evaluated at different time points. Additionally, the results obtained by implanting different films were compared with those of the no‐film implantation control group. All the procedures were approved by the Animal Research Committee of Jilin University (the ethical number: SY202309037).

At 4 and 8 weeks after surgery, we separately extracted the periodontal soft tissue around the second molar to assess the anti‐inflammatory activity of the E‐Janus Col/DS film. The expression levels of three typical pro‐inflammatory genes (IL‐1β, IL‐6 and IFN‐γ) in the tissue, were measured by real‐time PCR (Figure 7b). The results showed that at different time points, the expression of the above three pro‐inflammatory genes in the E‐Janus Col/DS film implantation group was remarkably lower than that in the control group without film implantation and also showed the lowest level compared with the other three groups implanted with various films. The results of the E‐Janus Col/DS film‐implanted group were lower than those of the E‐Janus Col/Cl‐ films (whose porous layer lacked DS), indicating the importance of the DS contained in the films for reducing inflammation. In addition, the group implanted with E‐Janus Col/Cl‐ films also had lower expression of IL‐1β and IL‐6 pro‐inflammatory genes than that implanted with E‐Col films (without porous layers), potentially indicating the beneficial contribution of the porous topology of the film for inflammation reducing. Besides, the E‐Janus Col/DS films implanted group also showed lower expression of genes associated with inflammation compared to the commercially available BioGide film implanted group. These results further demonstrate that E‐Janus Col/DS films can reduce periodontal tissue inflammation in vivo.

We further evaluated the regeneration of the defective AB below the second molar using micro‐computed tomography (CT) at 4 and 8 weeks postoperatively. The representative reconstructed 2D and 3D images are shown in Figure 7c. Two key indices of AB quality and morphology were analyzed using CT images, as shown in Figure 7d. The first was the quantitative bone mineral density (BMD) of the AB in each group at different time points after surgery, which can directly reflect the quality of AB reconstruction. The results showed that The E‐Janus Col/DS film‐implanted group had the highest AB density at different time points after surgery). The second is the semi‐quantitative distance (marked with dotted lines) between the alveolar bone crest (ABC) and the cementum junction (CEJ) after treatment, which can reflect the morphological reconstruction of the AB Generally, the smaller the ABC‐CEJ distance, the higher the AB height.

The results indicated that the rats implanted with the E‐Janus Col/DS film had a significant reduction in the ABC‐CEJ distance and an increase in the AB area, which was not observed in untreated animals. Besides, the group implanted with E‐Janus Col/DS films showed a smaller the ABC‐CEJ distance compared with the group implanted with BioGide commercial films. In summary, these results suggest that the implantation of the E‐Janus Col/DS film can provide a favorable ME for AB regeneration in vivo. Presumably, this was related to the anti‐inflammatory and osteogenic properties provided by the porous topology and loaded DS component of the E‐Janus Col/DS film.

**Figure 8:**
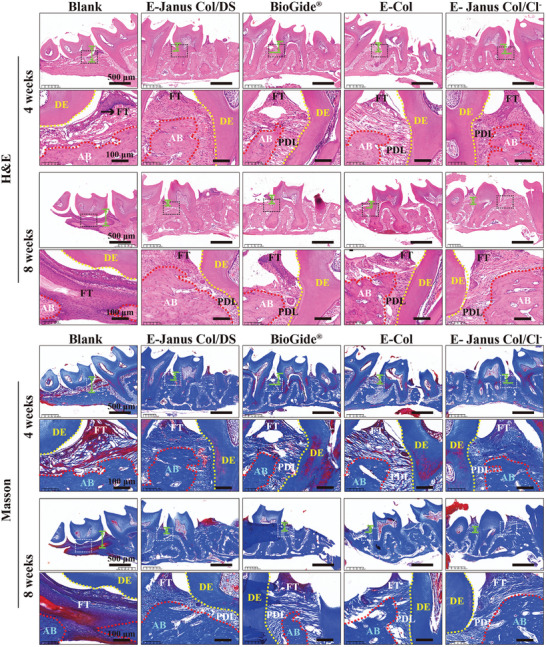
Histological observation of periodontal tissue. Representative H&E staining and Masson's trichrome (MT) staining images comparing the periodontal tissue regeneration in different groups after repair period of 4 and 8 weeks. (AB, host alveolar bone; DE: dentine; PDL: periodontal ligament; FT, fibrous; The distance between the cementum junction (CEJ) and the alveolar bone crest (ABC) marked with the green dashed line). (*n* = 4), scale bar: 500 and 100 µm for low‐power (2x) and high‐power (10x) objectives, respectively).

Histomorphological analysis using hematoxylin and eosin and Masson's trichrome staining was performed to observe newly formed periodontal tissues at 4 and 8 weeks postoperatively. The representative histological images in **Figure** **8** show the maxillary second molar in mice and its surrounding periodontal tissue. The enlarged images at the bottom distinguish the AB tissue (labeled AB), enamel (labeled DE), periodontal ligaments (labeled PDL), and gingival fibrous tissue (labeled FT). In the blank group with no film implant, AB tissue with a low height was observed below the DE, and FT tissues were found filling the large space between the AB and DE 4 weeks post‐operation. A large number of emerging inflammatory cells (blue area) and a smaller amount of AB tissue were observed after 8 weeks, indicating that AB absorption increased over time in cases of local inflammation due to periodontitis, while more FT tissues penetrated the area. These phenomena can lead to eventual tooth loss.

In comparison, the groups implanted with the four different films exhibited varying degrees of periodontal tissue regeneration and recovery of AB tissue height. Specifically, the group implanted with the E‐Janus Col/DS films displayed the highest levels of AB and complete periodontal tissue at both 4 and 8 weeks post‐operation. The AB tissue showed a partial height recovery and was tightly connected to the DE through the PDL tissue, whereas the FT tissue was located on the surface of the AB tissue without inward penetration. Furthermore, the AB tissue height and area in the group with the E‐Janus Col/DS implant were significantly higher than those in the other three groups implanted with commercially available BioGide films, E‐Janus Col/Cl films, and E‐Col films. (Note: Histological semi‐quantitative analysis of the distance between the CEJ and ABC is shown in Figure S19, Supporting Information). Histological immunofluorescence further qualitatively and quantitatively demonstrated that E‐Janus Col/DS had a higher number of M2 macrophages (fewer M1 macrophages) and osteoblasts at 4 and 8 weeks after implantation. (Figure S20, Supporting Information). These results suggest that E‐Janus Col/DS films have a superior capacity to promote the regeneration of AB tissue under periodontal pathological conditions. Presumably, this could be attributed to the immune regulatory functions endowed by the composition and microstructure of the E‐Janus Col/DS films (which reduced local tissue/cell pro‐inflammatory gene expression, as proved in Figure 7b; Figure S20, Supporting Information), meanwhile the featured composition and porous structure of the E‐Janus Col/DS films may also benefit osteoblast proliferation and differentiation, thereby accelerating the regeneration of AB tissue in an inflammatory environment.

## 3 Conclusion and Outlook

In summary, we present a controlled electroassembly method for collagen under the influence of salt, enabling the customization of collagen porous films with precise control over the porous structures, including porosity and pore size. The effects of different salt species with varying anion valences on the regulation range of the generated porous structures were investigated. Bioactive salt species such as the anti‐inflammatory drug DS have been used to induce and customize the porous structure of collagen films, allowing for DS loading and subsequent release from the films, thus further enhancing the functional composition of the film.

For the treatment of periodontitis, we employed the sequential electro‐assembly of collagen under preprogrammed DS conditions to fabricate Janus composite films with dense DS‐containing porous layers, enabling multifunctionality. The dense layer provides mechanical support and guides fibrous tissue growth while acting as a barrier to prevent tissue penetration into the bone defect. The porous layer containing DS facilitated osteoblast adhesion and provided a conducive three‐dimensional space for continuous proliferation. Moreover, the pathological environment of periodontitis is often marked by an excess of the M1 phenotype in macrophages, which release pro‐inflammatory cytokines that stimulate osteoclast formation and activity, leading to bone resorption. In contrast, M2 macrophages aid in later‐stage tissue repair by releasing anti‐inflammatory cytokines and bone formation factors (such as BMP‐2 and VEGF), thus fostering bone regeneration.^[^ ^7^, ^23^ ^]^ The DS‐containing porous layers delivered dual immunomodulator signals through the “physical signals” from the porous structure and the “molecule signals” from the gradually released DS. This combination inhibited the inflammatory phenotype of macrophages while promoting osteoblast differentiation (Presumably, osteoclast formation was also reduced by inhibiting the M1 phenotype of macrophages). In vivo studies using a mouse periodontitis model validated the beneficial properties of the film as it effectively inhibited the expression of pro‐inflammatory genes in cells/tissues and facilitated the healing of periodontal tissues.

Electrofabrication is an emerging additive manufacturing method with simple, fast (<20 min), and safe (<10 V) features that provide unprecedented microstructure control capabilities. Our study demonstrates the potential of the salt effect in regulating the electroassembly of biomacromolecules to achieve specific biological functions. Considering the convenient input of electrical signals and the mild, modifiable aqueous reaction system, electrofabrication is a simple, fast, and programmable manufacturing technology. This method has enormous potential for expanding the new biological functions of well‐established biomedical polymers (e.g., collagen), allowing the assembly of customizable compositions and microstructures to tailor the specific needs of regenerative medicine.

However, it's important to acknowledge the limitations of our study. Echoing many reports in the literature, we observed that the porous structure of electro‐assembled collagen films can modulate immune responses. Yet, the underlying biological mechanisms, such as the effect on macrophage polarization and the influence of the material matrix on immunoregulation, need more exploration. Additionally, detailed studies of the dose‐effect relationship between porous structural features, such as porosity and pore size, and their immunomodulatory effects are needed. Given collagen's potential as a versatile material in tissue engineering, regenerative medicine, and implantable medical devices, a deep understanding of the biological impacts of its porous topology on immune regulation is crucial for both clinical and practical applications.

## 4 Experimental Section

More details are provided in the Supporting Information

## Conflict of Interest

The authors declare no conflict of interest.

## Supporting information

Supporting Information

Supporting Information

Supporting Information

## Data Availability

The data that support the findings of this study are available from the corresponding author upon reasonable request.
