# Supplementary material for: Programmable Electro‐Assembly of Collagen: Constructing Porous Janus Films with Customized Dual Signals for Immunomodulation and Tissue Regeneration in Periodontitis Treatment
Source: Adv Sci (Weinh). 2024 Jan 8;11(13):2305756. doi: 10.1002/advs.202305756 (PMC10987108; doi:10.1002/advs.202305756)
Supplement: Supplementary file 1 — Supporting Information [file ADVS-11-2305756-s003.pdf]

ADVANCED SCIENCE
Open Access

Supporting Information

for *Adv. Sci.*, DOI 10.1002/advs.202305756

Programmable Electro-Assembly of Collagen: Constructing Porous Janus Films with Customized Dual Signals for Immunomodulation and Tissue Regeneration in Periodontitis Treatment

Miao Lei, Haoran Wan, Jia Song, Yanhui Lu, Ronghang Chang, Honglei Wang, Hang Zhou, Xuehui Zhang, Changsheng Liu and Xue Qu**

Supporting Information

Programmable Electro-assembly of Collagen: Constructing Porous Janus Films with Customized Dual Signals for Immunomodulation and Tissue Regeneration in Periodontitis Treatment

Miao Lei [1‡], Haoran Wan [1‡], Jia Song [2‡], Yanhui Lu [2‡], Ronghang Chang [1], Honglei Wang [1], Hang Zhou [1], Xuehui Zhang [2,*], Changsheng Liu[1,] and Xue Qu [1, 3, 4] *

1 Key Laboratory for Ultrafine Materials of Ministry of Education, Frontiers Science Center for Materiobiology and Dynamic Chemistry, School of materials science and engineering, East China University of Science and Technology, Shanghai 200237 (China)

2 Department of Dental Materials & Dental Medical Devices Testing Center, NMPA Key Laboratory for Dental Materials, Peking University School and Hospital of Stomatology, Beijing, 100081, (China)

3 Shanghai Frontier Science Research Base of Optogenetic Techniques for Cell Metabolism, East China University of Science and Technology, Shanghai 200237 (China)

4 Wenzhou Institute of Shanghai University, Wenzhou 325000 (China)

‡ These authors contributed equally to this work.

* Corresponding author emails: quxue@ecust.edu.cn; zhangxuehui@bjmu.edu.cn

This PDF file includes:

Experimental Section
Figs. S1 to S20
Legends for movies S1 to S2

Other Supplementary Materials for this manuscript include the following:

Movies S1 to S2

Experimental Section

Materials and Devices: Acid-extracted Type I collagen from porcine skin was procured from Haohai Biotechnology Co., Ltd. (China). Acetic acid、hydrogen peroxide、NaCl、Na_2SO_4、Na_3Cit were purchased from Shanghai Lingfeng Chemical Reagent Co., Ltd. Diclofenac sodium (Ds) was purchased from Shanghai Macklin Biochemical Technology Co., Ltd. Simulated human salivary (SHS) was purchased from Shanghai Yuanye Bio-Technology Co., Ltd. Collagen was purified to remove insoluble impurities and dialyzed ($M_{w\ cutoff}$ = 7 kDa) to remove soluble salts before use. The ultrapure water used was prepared in a two-stage Milipore Milli-Q Plus purification system. All reagents related to cell experiments were purchased from Gibco (Grand Island, NY). Electrodeposition was performed using a three-electrode system in which a titanium sheet (dimensions 2.5 cm × 2.5 cm) was used as the working electrode (cathode), a platinum wire was used as the counter electrode (anode), and an Ag/AgCl electrode was used as the reference electrode.

In Situ Visual Monitoring of Electrodeposition in Fluidic Channel: A titanium sheet and a platinum sheet with a thickness of 0.05 mm were used as the working electrode and the counter electrode, respectively, and then fixed on a quartz glass plate to form micro-channels with a spacing of 1.5 mm. The working area of both electrodes is 2 mm × 2 mm.[1] The electrodes at both ends are respectively connected to the electrochemical workstation (CHI 660e) by wires. The electrolyte solution was added to the microchannel, and the electrodeposition was performed at a current density of 6.67 mA/cm2, while the growth of the hydrogel was observed and recorded using an inverted microscope.

Electro-fabrication of Collagen-Based Materials: Collagen solution (1%, w/v; pH 3.5) was prepared by dissolving type I collagen in acetic acid. Different amounts of salts (NaCl, Na_2SO_4, Na_3Cit, and diclofenac sodium) were added to the collagen solution for electrodeposition of collagen with soluble salts. Then, 0.1M H_2O_2 was added and stirred evenly to obtain the electrolyte ready for use. Electrodeposition at a specified current density (6.67 mA cm^{-2}) and time (1000 s) of collagen or a mixed solution of collagen and soluble salts. After electrodeposition, the gel-coated titanium sheet was rinsed three times with ultrapure water. The gel material was stripped from the cathode surface and subsequently subjected to freeze-drying for further characterization. The glutaraldehyde (GA) cross-linking process was performed by soaking dry films in GA solution (1% w/v, 90% v/v ethanol-water solution) for 15 min. And, the cross-link films were repeatedly washed with ultrapure water to remove the residual riboflavin or GA for subsequent tests.

Characterization of Films: The viscoelastic behavior of the hydrogel films was investigated using a rotational rheometer (MARS III, HAAKE). Scanning electron microscopy (S-4800, Hitachi) was used

for the morphology analysis. Energy-dispersive spectroscopy (QUANTAX 400-30, BRUKER AXS) was used to measure the elemental distribution of the films. Zeta potential of collagen molecules were measured using a Nano ZS Zeta meter (Malvern). Chemical analysis and crystalline phases of the lyophilised films were determined using an ATR-FTIR spectrometer (Nicolet 5700, Thermo) and an X-ray diffractometer (Rigaku D/max2550 VB/PC, Bruker). Image Pro 5.0 software (Media Cybernetic, USA) was used to statistically estimate the pore size distribution from surface images of thin films. The total porosity of the film was estimated gravimetrically,[2] and the apparent density and porosity of the film were calculated using equations (1) and (2),[3] respectively:

$$\text{Apparent density } (g/cm^3) = \text{Mass of Film (g)} / [\text{Film thickness (cm)} \times \text{Film area } (cm^2)] \quad (1)$$

$$\text{Porosity } (\%) = [1 - \text{Apparent density } (g/cm^3) / \text{Bulk density } (g/cm^3)] \times 100\,\% \quad (2)$$

The dynamic biomechanical testing instrument Electro-320 (TA, USA) was used to measure the mechanical properties of the film (strip sample: 10 mm × 20 mm). For the cyclic tensile test, the film was first cut into dumbbell shaped samples of 20 mm length and 1 mm thickness, and the cyclic tensile test was conducted at a speed of 0.04 N s^{-1} for 10 cycles. The cross-linked films were immersed in either simulated human salivary (SHS) or phosphate buffered saline (PBS) and incubated at 37°C for 7 days. Afterward, their tensile properties were tested. And the hydrophilicity test of the surface of the film material was carried out using a contact angle tester (JC2000D2).

Water content measurement: We measured the water content of thin film hydrogels by comparison of weight before and after lyophilisation. Excess water was wiped off the surface of the film gel and the gel samples were immediately frozen in liquid nitrogen and then lyophilized. The weight before (M_w) and after freeze-drying (M_d) was measured using an electronic balance. The water content was calculated as $[(M_w - M_d)/ M_w] \times 100\%$.[4] The quantitative swelling ratio of the Janus porous film: Specifically, the dry Janus film was first immersed in PBS solution for 2 hours to reach a saturated moisture state. Surface moisture was gently removed with filter paper, and the initial weight (W_0) of the wet film was weighed and recorded. Subsequently, the wet film was returned to the PBS solution and incubated at 37℃ until designated time points (1 day, 3 days, 5 days and 7 days), followed by reweighing and recording the weight (W_t) of the wet film. Finally, the swelling ratio at different time points was calculated using the formula [Swelling ratio (SR)=$(W_t-W_0)/W_0 \times 100\%$].

Drug release assessment: Drug release measurements were performed by using a UV/Vis-spectrophotometer (UNICAM UV 540, Thermo Spectronic, Cambridge, UK). Preparation of standard curve for DS release study[5]: the standard curve for diclofenac sodium (DS) was prepared prior to drug study release. DS was dissolved into PBS buffer solution (pH=7.4) and diluted to different concentrations. The UV absorbance of the DS solution was recorded, and the calibration curves were

drawn based on the UV absorbance at 276 nm and different concentrations of the DS solution. The curve exhibits linear behavior over the entire concentration range. A series of DS mixed collagen solutions with different concentrations were prepared for electrodeposition. After the deposition was completed, the gel material coated with a titanium sheet should be washed with ultrapure water. The gel material should then be carefully removed, and the quality of the gel material should be recorded. The remaining electrolyte was collected, and the concentration of residual diclofenac sodium was measured by a UV-visible spectrophotometer, and then the encapsulation efficiency of the drug was calculated. Calculated according to the following formula (there are three parallel samples in each group, and the average value is taken):

$$\text{Drug Encapsulation Efficiency (\%)} = \frac{\text{total amount of drug-amount of unbounded drug}}{\text{total amount of drug}} \times 100\ \%$$

In order to study the release kinetics of diclofenac sodium, before the start of the experiment, the film material was cut into a size of 1 cm × 1 cm, and then placed in a 6-well plate, and 10 mL of PBS solution was added to each well to ensure that the material was completely submerged. After the orifice plate was sealed, it was placed on a constant temperature bed at 37°C. Samples were taken at a fixed time, 2 mL each time, and 2 mL of fresh PBS solution was added at the same time. The absorbance of the solution was measured using a UV-Vis spectrophotometer, and the corresponding concentration was calculated from the standard curve. The change in drug concentration over time was measured to obtain the curve of cumulative drug release in the material over time; at the same time, the release of the drug per unit area of the material was calculated. The cumulative drug release rate and the release rate per unit area can be calculated according to the following formula (there are three parallel samples in each group, and the average value is taken):

$$W_C=\frac{C_{ti}\times V+\sum_{t=0}^{t=i-1} C_t\times V_t}{m}\times 100\%$$

$$W_P=\frac{W_C}{S}\times 100\%$$

In the formula, W_C is the cumulative release amount; C_t and V_t represent the concentration (mg cm^{-3}) and volume (cm^3) of the target drug in the solution taken out at t=i-1, respectively; C_{ti} represents the concentration of the solution at t=i (mg cm^{-3}), V represents the total volume of the solution (cm^3).

In Vitro Antibacterial Studies: Gram-positive Staphylococcus aureus (S. aureus) was chosen as the bacterial model. Five groups of films were used for the antibacterial test: (1) dense collagen film (D-col), (2) porous collagen film (P-Col), (3) Janus collagen film (Janus-Col/DS), (4) blank filter paper (completely soaked in 5 mg/mL vancomycin solution), (5) blank filter paper (completely soaked in physiological saline). The films were cut into 6mm diameter circles and placed in 48-well plates. Subsequently, each sample well is supplemented with 500 μL of bacterial suspension (S. aureus, 10^6 CFU mL^{-1}). After incubation at 37 °C for 18 h, bacterial numbers were obtained by using dilution plate counting. Antibacterial efficiency was calculated using the following formula:

$$\text{Antibacterial efficiency (\%)} = [(C-M)/C] \times 100\%$$

where C is the number of bacterial colonies on the blank group and M is the number of bacterial colonies on the film.

The inhibition zone assay: The film were made into disc shapes with a diameter of 6 mm, which were placed onto the agar plates containing 100 μL of bacterial suspension (S. aureus, 10^6 CFU mL^{-1}), and then incubated for 18 h at 37 °C to observe the growth of bacteria.

In Vitro Cell Studies: For the cell barrier assessment, the films were cut into 20mm diameter circles. They were then sterilized, fixed on Cell Crown™ (Sigma), and placed in 24-well plates with no contact to the bottom of the wells. Suspend mouse fibroblasts (L929) in the culture medium at a density of 5.0 × 10^4 cells/ml. After incubating for 1, 3 and 5 days, live/dead staining (DOJINDO) was carried out on the cells. The distribution of cells on the film was observed using a confocal microscope (CLSM, Nikon A1R).

For the cell viability and proliferation tests, the films were cut into circles of 10mm diameter, and placed in a 48-well plate. MC3T3-E1 were seeded and cultured on the films at 5 × 10^4 cells per well. Cell morphology was evaluated by scanning electron microscopy (SEM), and cell proliferation rate was evaluated by CCK-8 assays (DOJINDO) after 1, 3 and 5 days of culture.

For macrophage adhesion to material surfaces, macrophages (LPS stimulation for 2 h at a concentration of 10 μg mL^{-1}) were inoculated on the material surface at 5 × 10^4 cells per well, cultured for 24 hours and then fixed with glutaraldehyde, and the cell morphology was assessed by scanning electron microscopy (SEM).

To evaluate the cytotoxicity of the films, their extracts were used in this study. The films were immersed in culture medium (DMEM) at 37°C for 24 hours. After aspirating the medium, 10% (v/v) fetal bovine serum (FBS, Gibco) was added. Fibroblasts (L929, CM-2104) obtained from ATCC were then inoculated into 96-well plates at a density of 6 × 10^3 cells/mL per well. After 12 hours, the medium was replaced with either extract or fresh DMEM medium supplemented with 10% (v/v) fetal bovine serum (positive control). The cells were then co-cultured for 1, 3, and 5 days. Cell viability was assessed using CCK-8 solution at the indicated time intervals.

For anti-inflammatory performance tests, mouse-derived macrophages (RAW 264.7) were purchased from the American Type Culture Collection (ATCC), and cultured in DMEM (Gibco) medium containing 10% FBS (Gibco) and 1% penicillin in a cell culture incubator at 37°C (5 % CO_2). When the cell growth density is high, passaging according to 1:4. Before the experiment, macrophages were stimulated with lipopolysaccharide (LPS-10 μg mL^{-1}) for 2 h to activate macrophages. The material was then cut into discs with a diameter of 10 mm, placed in a 48-well plate, and sterilized by irradiation. Then macrophages were seeded on the material and cultured in a 37°C cell culture incubator (5 % CO_2) for 24 h. After culturing for 24 h, the cells were harvested, and total RNA was extracted with Trizol

reagent. Using quantitative real-time PCR to determine mRNA expression. The expression of inflammatory factors in each group was detected as described above, with GAPDH as the internal reference, and the main primer sequences are listed in the table below. The RAW264.7 activated by lipopolysaccharide (LPS, 10μg mL^{-1}) for 2h were seeded onto the surfaces of different films with a density of 10^5 cells, then cells were lysed after 24 hours culturing for detecting the expression of the M1 and M2 phenotypes related genes.

The flow cytometry assay is used to more clearly confirm the effects of films in different groups on the polarization of macrophages (i.e., RAW 264.7). Briefly, the RAW 264.7 cells were co-cultured with different groups of films (TCP was set as blank group) for 24 hours. After washing with PBS twice, cells were re-suspended with cell staining buffer. Subsequently, the cells were then stained with FITC rat anti-mouse CD86 and APC rat anti-mouse CD206 to determine M1 and M2 phenotypes by flow cytometry, respectively. The samples were analyzed on an Accuri C6 flow cytometer (BD) and data were analyzed using Kaluza analysis software (Beckman Coulter).

Table Sequences of primers

GAPDH

Forward primer: GCAAGGACACTGAGCAAGA

Reverse primer: GGATGGAAATTGTGAGGGAG

TNF α

Forward primer: CTCTTCTCATTCCTGCTTGTG

Reverse primer: GGGAACTTCTCATCCCTTTG

iNOS

Forward primer: CAGACACATACTTTATGCCACC

Reverse primer: GTCATGTTTGCCGTCACTC

CD 206

Forward primer: TGAGGGAAGCGAGAGATTATG

Reverse primer: CCAGGTTAAAGCAGACTTGG

ARG-1

Forward primer: GTCATTTGGGTGGATGCT

Reverse primer: GAAAGGACACAGGTTGCC

For the cell osteogenic differentiation tests, the films were cut into circles with a diameter of 10 mm and placed in a 48-well plate. Macrophages (LPS stimulated for 2h) and osteoblasts were mixed at a ratio of 4:1 and seeded on the material.[6] After 14 and 21 days of osteogenic incubation, the medium was removed and the cells were washed with PBS. Then, 250 μL of 1% Nonidet P-40 (Beyotime) was added to each well, and incubated at 37°C for 1 h. 50 μL of the obtained cell lysate was added to each well of a 96-well plate, followed by the addition of 50 μL of p-nitrophenyl phosphate (Sangon)

substrate solution. The mixed solution was then incubated at 37°C for 1 h. ALP activity was quantified using a microplate reader at a wavelength of 405 nm. The activity of ALP was expressed as OD value at 405 nm/mg of total protein amount. The total protein concentration was determined using the BCA assay kit (Beyotime). ALP staining was performed using the BCIP/NBT ALP staining kit. Briefly, macrophages and MC3T3-E1 cells were seeded on the material at a ratio of 4:1 and cultured for 14 days. The cells were fixed with 2.5% glutaraldehyde and subsequently incubated with a mixture of nitro blue tetrazolium and 5-bromo-4-chloro-3-indolyl phosphate. For the alizarin red (ARS) staining, after 21 days of osteogenic induction culture, Cells were fixed with 4% paraformaldehyde for 10 min, and then an appropriate amount of alizarin red staining solution was added and incubated at room temperature for 30 min. Samples were washed with ultrapure water to remove unbound dye.

In Vivo Studies: All animal experiments adhered to the NIH guidelines for the care and use of laboratory animals (NIH Publication no. 85-23 Rev.1985) and were approved by the Animal Care and Use Committee of Jilin University (the ethical number: SY202309037). For subcutaneous implantation experiments, to assess the histocompatibility of thin film materials and observe how surrounding cells and tissues penetrate these thin films in vivo, a total of 18 SD rats (male, body weight 180 ± 5 g) were used. Films with a circular shape (a diameter of 6 mm) were sterilized using ethylene oxide gas (note: all films crosslinked by 0.5% w/v glutaraldehyde for 15 min in all in vivo experiments). The SD rats were randomly divided into three groups for different films' implantation. In order to alleviate the pain and panic of animals, anesthesia was carried out using sevoflurane by inhalation. The dorsal skin was shaved and disinfected with povidone iodine, and a 1.5 cm long incision was made using a blade mounted on a scalpel, followed by scissors to create two subcutaneous pockets, different samples were gently inserted into the formed pockets individually, and the incisions were sutured with 3-0 non-absorbable nylon sutures. After 1, 4 and 12 weeks of implantation, respectively, the animals were subjected to euthanasia, and the implanted films were retrieved for gross observation and histological analysis. The H&E-stained and IHC-stained tissue samples were observed under optical microscopy and inflammation reactions, including cell and tissue invasion, were analyzed.

To perform a periodontitis model, according to previous studies, we used silk ligation and bacterial infection to induce periodontitis in mice.[7] The animals were divided into five groups (eight mice per group): (1) no-film treated, (2) E-Col film, (3) E-Janus Col/Cl^- film, (4) E-Janus Col/DS film, and (5) BioGide® film. Briefly, the eight-week-old male C57BL/6 mice were initially subjected to a standard diet and maintained under specific pathogen-free (SPF) conditions for one week to facilitate environmental adaptation. Anesthesia was induced in the mice by intraperitoneal injection of 1% pentobarbital. Subsequently, a type 5-0 silk thread was used to create a circumferential ligation and knot around the neck of the maxillary second molars of the mice. A suspension of Porphyromonas

gingivalis (10^9 CFU mL^{-1}) was injected daily into the silk-ligated molar site. After a ten-day period following ligation, the establishment of periodontal pockets was observed. After confirming that the mouse periodontitis model was successfully established, the ligature was removed, and the mice were randomly divided into two groups, which were treated for different periods of time, 4 weeks and 8 weeks. First, the palatal part of the upper second molar of the mouse was treated with the jaws of the micro-tweezers from the gingival sulcus on both sides of the neck to perform palatal gingival flap treatment, and the flap was raised to place the material (strip sample: 2 mm × 4 mm) on the palatal side of the upper second molar. The palatal gingival suture suspension was fixed to the teeth with 7-0 sutures, and the sutures were removed one week later. Four and eight weeks post-implantation, the animals were sacrificed, and the amounts of bone regeneration at defect sites were evaluated using μCT analysis. All the specimens were standardized, and μCT images were calibrated for proper comparative analysis. The vertical bone loss at each defect site was evaluated by measuring the distance between cementoenamel junction (CEJ) and alveolar bone crest as well as bone mineral density (BMD) ac-cording to methods reported in the literature. For histological analysis, after micro CT analysis, the maxilla was decalcified by 0.5 M ethylene diaminetetraacetic acid (EDTA) for two weeks, dehydrated with graded ethanol, embedded in paraffin blocks, and cut into 5 μm thick sections at the defect site, and then the tissue sections were processed with H&E staining and Masson trichrome staining, and visualised with a light microscope for further assessment of alveolar bone repair. For immunofluorescence analysis, antibodies against OCN(Bioss, bs-4917R)、CD86 (Proteintech, 13395-1-AP) and CD206 (Abcam, ab64693) were utilized to assess the inflammatory response and newly-formed bone tissues. All antibodies were used according to the manufacturer's instructions. Quantitative analysis was performed using Photoshop software (V.2020).

Periodontitis is a chronic inflammatory disease that affects the regeneration of periodontal tissue. In order to evaluate the effect of the composite film implanted in the mouse periodontal defect to reduce the inflammatory response, the inflammatory cytokines in the tissue around the defect were measured to judge the therapeutic effect of the composite film. The specific experimental procedure was as follows: the buccal and palatal tissues of maxillary molars were dissociated and dissociated using collagenase and DNase I at 37°C for 15 minutes, and then the enzymes were inactivated by adding EDTA. Next, the extracted tissue was crushed to obtain cells, and the levels of

inflammation-related factors (IL 6, IL 1β, IFN γ) were measured by real-time quantitative PCR.

Statistical Analysis: All data are presented as mean ± standard deviation (SD) of triplicates unless otherwise indicated. Statistical analyses were performed Origin (version 8.0) software using a two-tailed Student's t-test for two-sample comparison or one-way ANOVA with Tukey's multiple comparisons post hoc test. In these analyses, significant differences were accepted at *p < 0.05, **p < 0.01, ***p < 0.001.

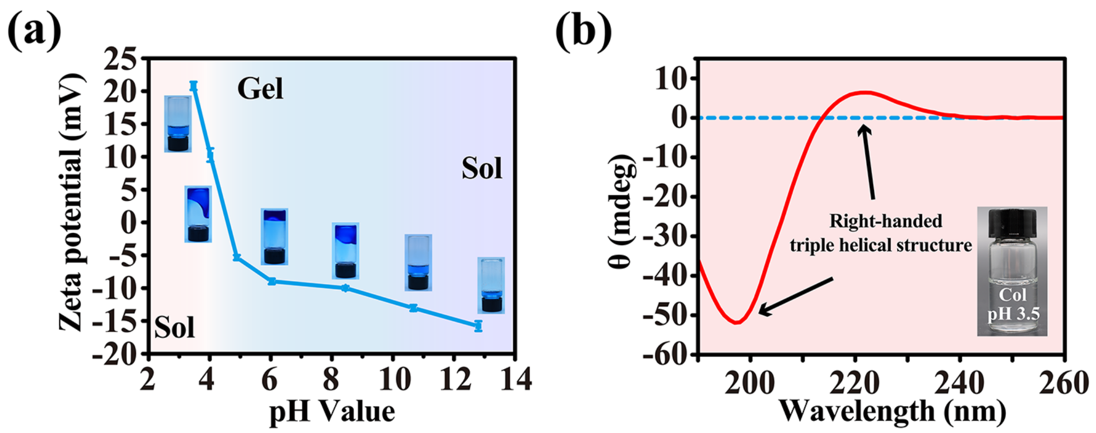

Fig. S1. Characterization of acid-soluble type I collagen. (a) Zeta potential (n=3) and (b) circular dichroism (CD) spectrum of Collagen I. The zeta potential of collagen molecules (1 mg mL^{-1}) at different pH values was analyzed using a Nano ZS zeta sizer (Malvern). The results show that the isoelectric point (IP) of collagen I ranges from 4.5 to 5, which indicates the pH range at which the net charge of collagen I becomes neutral. In addition, a clear sol-gel phase transition phenomenon can be observed. CD spectrum of collagen solution (0.125 mg mL^{-1}) was obtained using a JASCO J-815 spectrometer (1 mm diameter quartz cell). The CD results show that collagen I still maintains a right-handed triple helix structure under acidic conditions (pH=3.5), which is the same as the pH of the electrolyte.

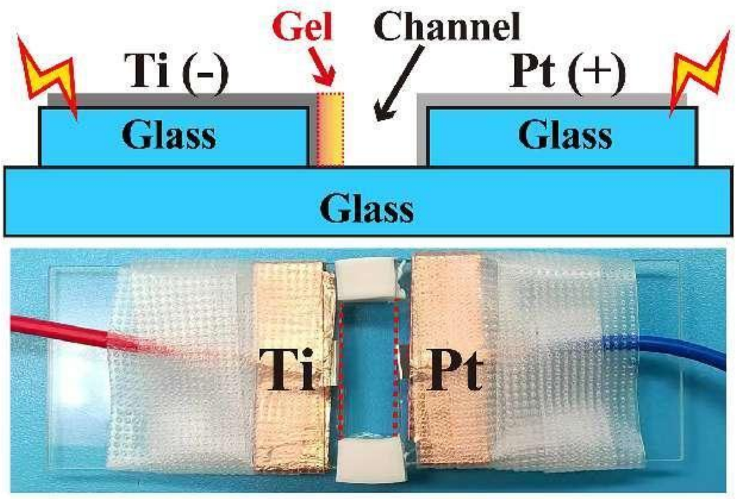

Fig. S2. In situ imaging of electrodeposition within a fluid channel allows direct observation of the emergent microstructure (see **Movies S1** and **S2** in the Supporting Movies).

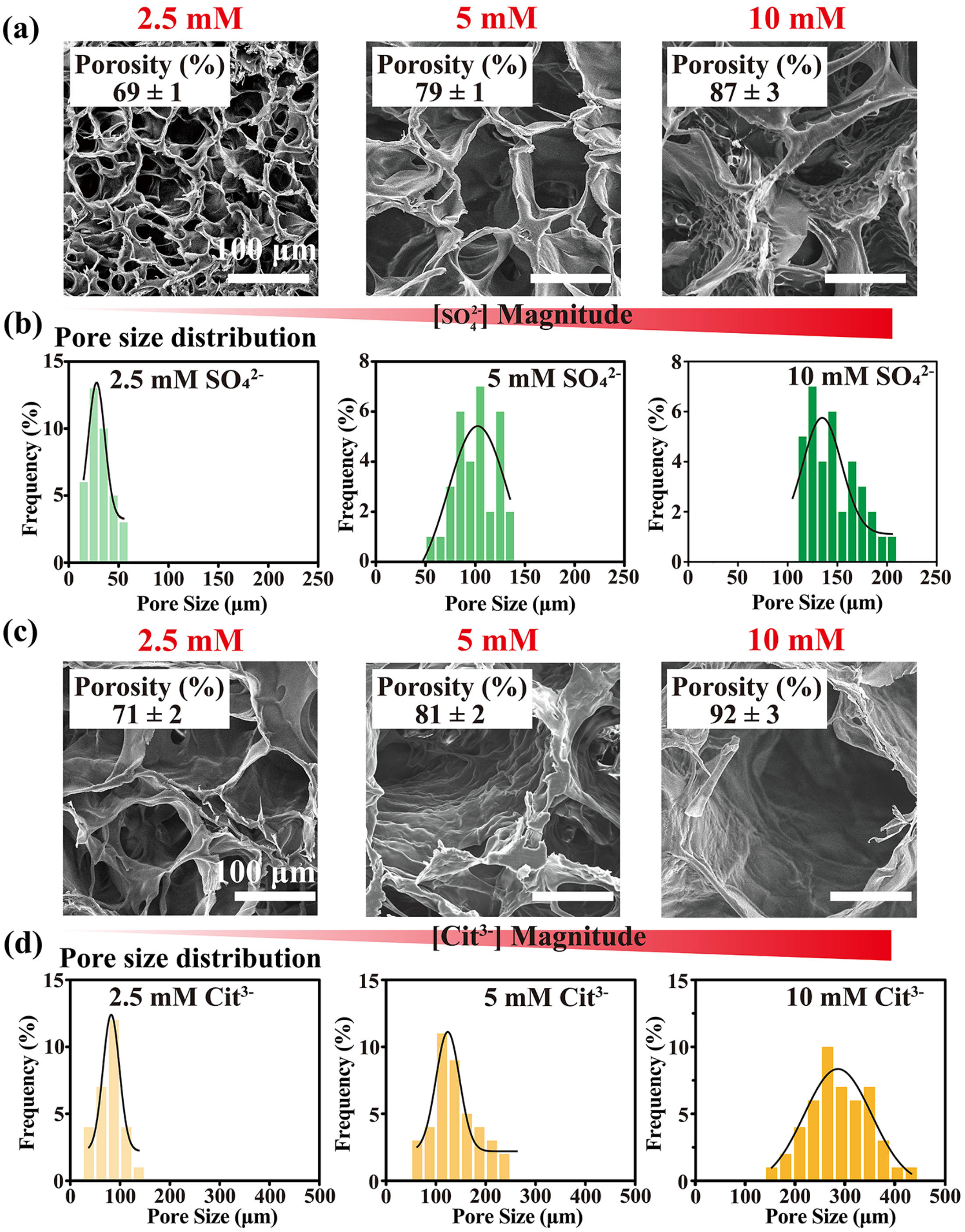

Fig. S3. Tuning the electrofabricated film porous topology by programming the concentration of (a) sodium sulfate and (b) sodium citrate. SEM images in (c) show the topology of the collagen film surface. Changing soluble salt concentration can be used to tune porosity. (d) Histogram of pore size distribution on the surface of collagen films (n=100), prepared with different salt species and varying concentrations.

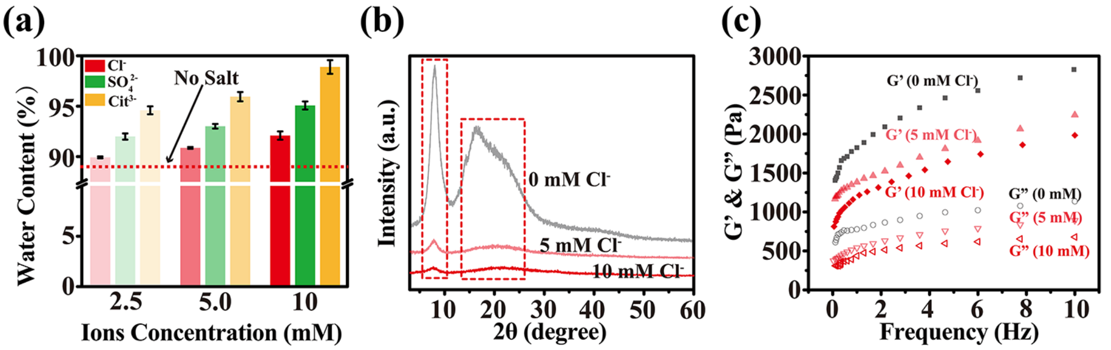

Fig. S4. (a) The water content of the electro-assembled collagen films in the presence of varying amounts of different salts species. (b) XRD patterns show that the addition of NaCl reduces the crystallinity of the freeze dried collagen film, and also reduces (c) the storage modulus of collagen hydrogel films.

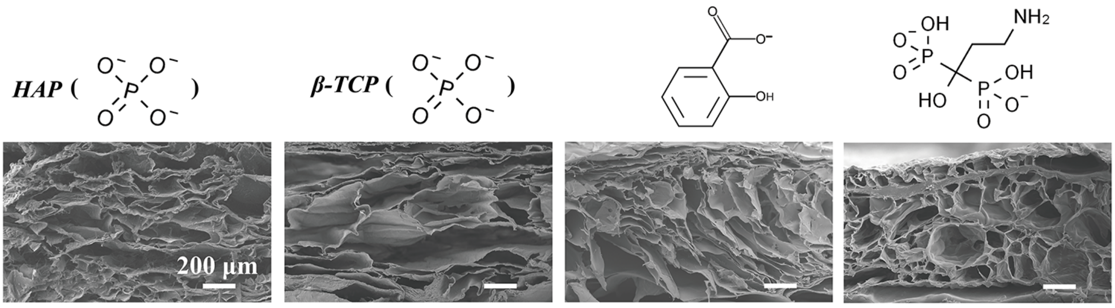

Fig. S5. SEM cross-sectional image of freeze-dried collagen film prepared by adding calcium phosphorus salts (i.e. HAP and TCP) and different kinds of ionic drugs (i.e. salicylate and pamidronate).

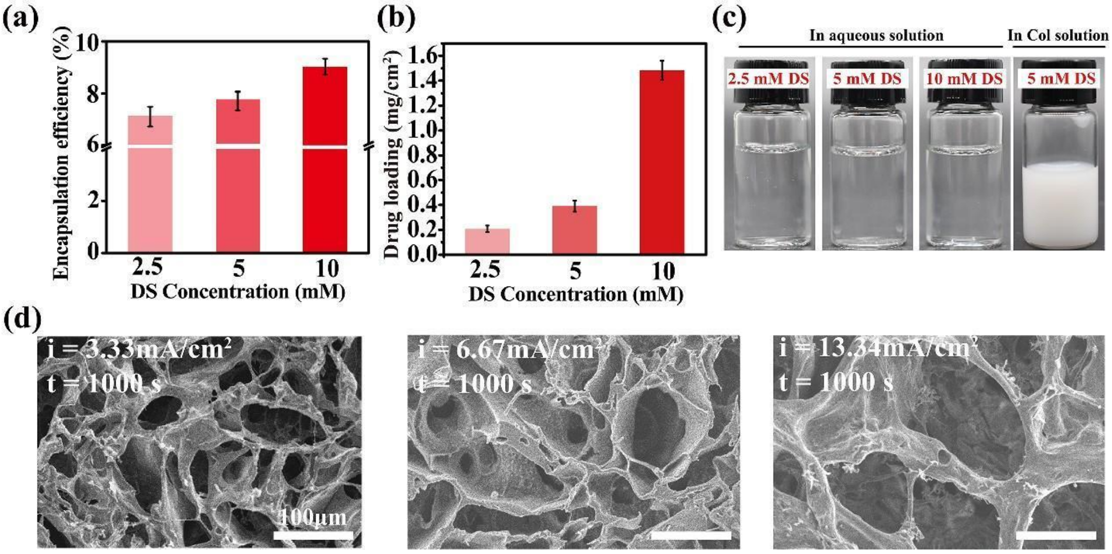

Fig. S6. (a) DS encapsulation efficiency and (b) the maximum DS loading of the electro-assembled collagen films under varying DS concentrations. (n=4). (c) The optical images show the aqueous solution of DS in the concentration range of 2.5 mM to 10 mM presents a transparent appearance, and the collagen solution containing 5 mM of DS has a milky appearance. (d) The SEM images show the surface morphology of freeze dried E-Col/DS films (the

concentration of DS is 5 mM) prepared using varying current densities.

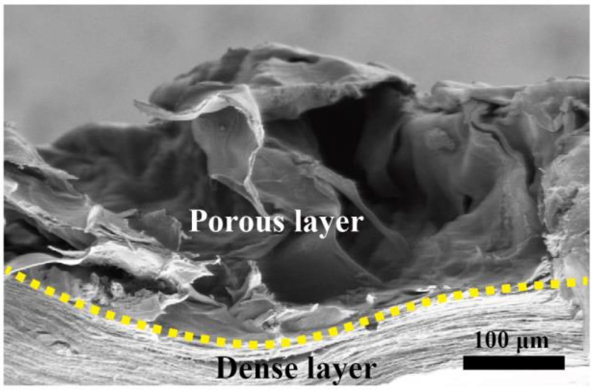

Fig. S7. The fracture cross-sectional SEM image of E-Janus Col/DS film after stretching, indicating that the porous layer of the Janus film was locally damaged but remained relatively closely bond to the dense layer without separation.

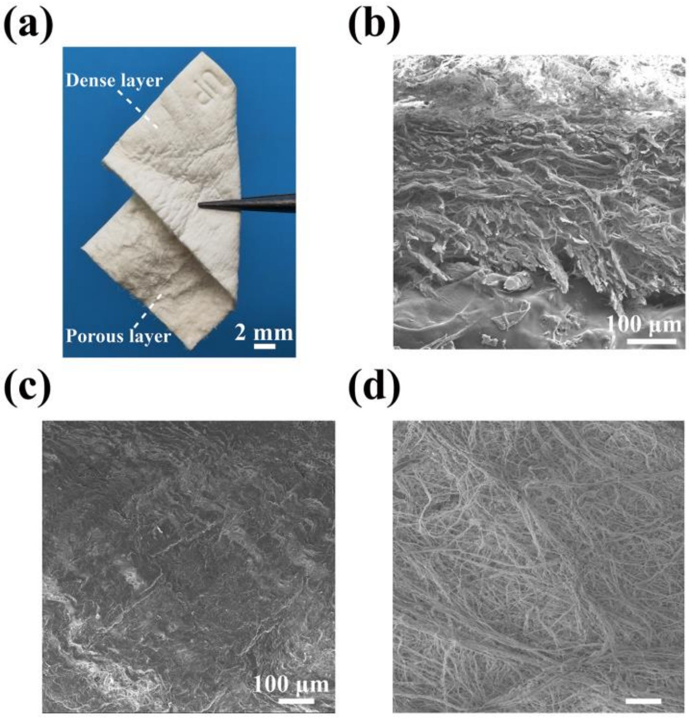

Fig. S8. Structural characterization of the BioGide® films. (a) Optical image of the BioGide® film. (b) The cross-sectional SEM image of BioGide® film, presenting the typical bilayer structure composed of a dense layer and a porous layer. The SEM images of (c) smooth surface and (d) rough surface of the BioGide® film.

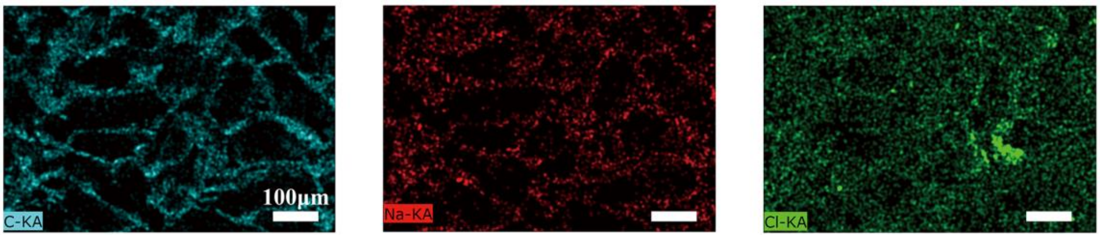

Fig. S9. Elemental mapping of C, Na and Cl elements of the porous face of the E-Janus Col/DS film.

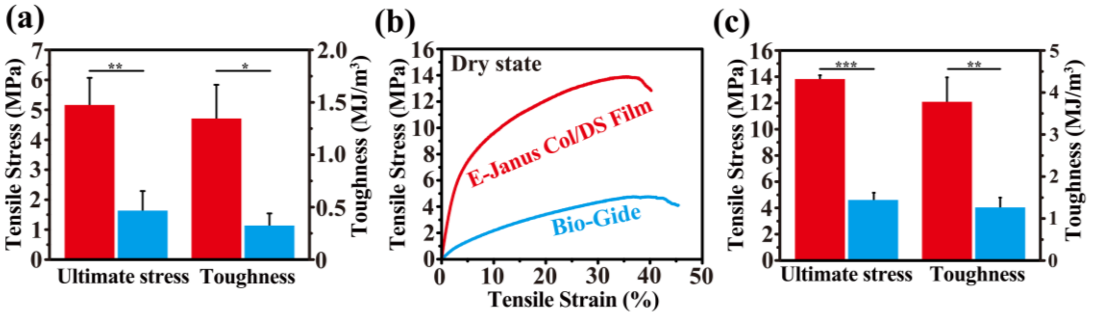

Fig. S10. (a) Comparison of the quantitative tensile strength and toughness of the wet E-Janus Col/DS films and the BioGide® films. (b) The representative stress-strain curves of dry E-Janus Col/DS film and BioGide® film. (c) Comparison of the quantitative tensile strength and toughness of the dry E-Janus Col/DS films and the BioGide® films. (n=3). (* $p < 0.05$, ** $p < 0.01$ and *** $p < 0.001$; All data are presented as mean ± SD. One-way ANOVA was used for comparison).

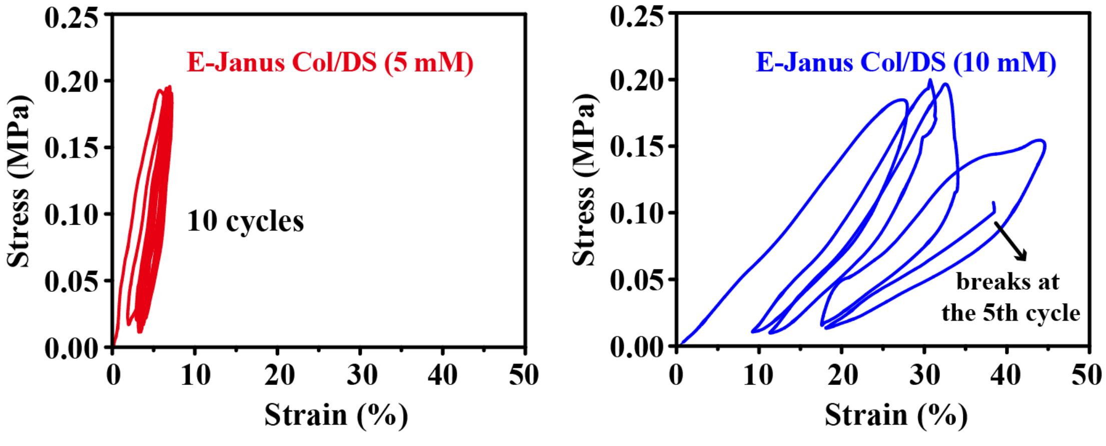

Fig. S11. The representative cyclic loading-unloading curves of wet E-Janus Col/DS films with 5 mM and 10 mM DS fabricated porous layer, which indicating the E-Janus Col/DS film with 5 mM DS fabricated porous layer exhibits better mechanical stability, no fracture, mechanical degradation and significantly irreversible deformation were observed during cyclic loading. While, the E-Janus Col/DS film with 10 mM DS fabricated porous layer exhibits reversible deformation and breaks at the 5th cycle of loading-unloading.

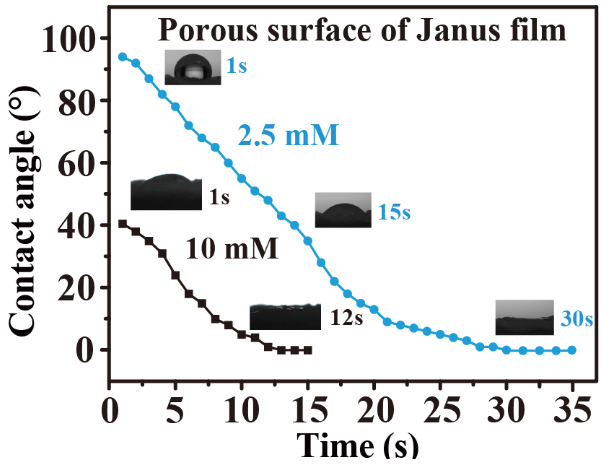

Fig. S12. Dynamic water contact angle measurements for the porous faces of the Janus Col/DS film fabricated with 2.5 mM and 10 mM DS concentration demonstrate the droplet placed on the porous surface of the Janus film fabricated by 2.5 mM DS needs 30 seconds to fully spread. In contrast, the dynamic water contact angle of droplets placed on the porous surface of Janus films fabricated by 10 mM DS rapidly decreases within 12 seconds. Presumably, the larger pore size and higher porosity of Janus film's porous layer contribute to the spreading and infiltration of the moisture.

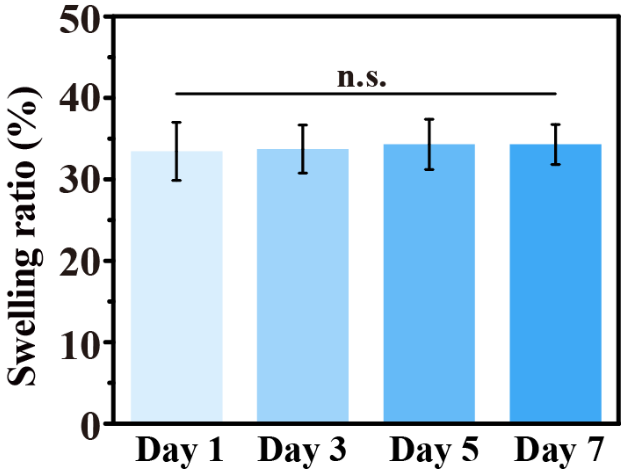

Fig. S13. The saturated swelling ratio of E-Janus Col/DS film at different time point, indicating the Janus porous film did not show excessive swelling during 1 week's incubation (n = 3).

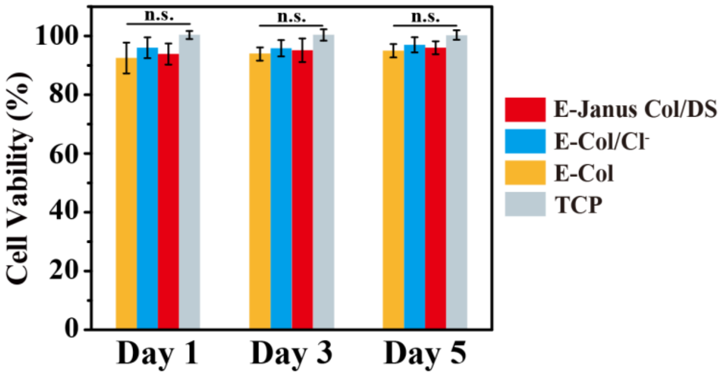

Fig. S14. CCK-8 assay of L929 cells after 1, 3 and 5 days culturing with the immersed DEME of different films. The results indicate that all films demonstrated good biocompatibility without cytotoxicity (n=3). [Note: 100% is normalized to the cell viability on the tissue culture polystyrene plate (TCP)].

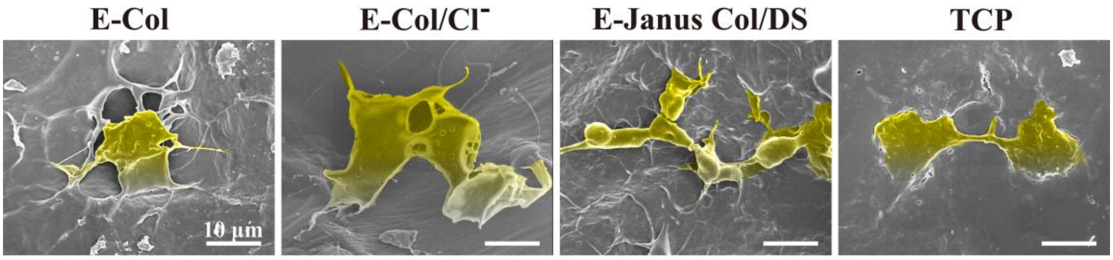

Fig. S15. SEM images of RAW 264.7 cells (marked in pseudo-yellow color) adhering to the surfaces of different films and TCP.

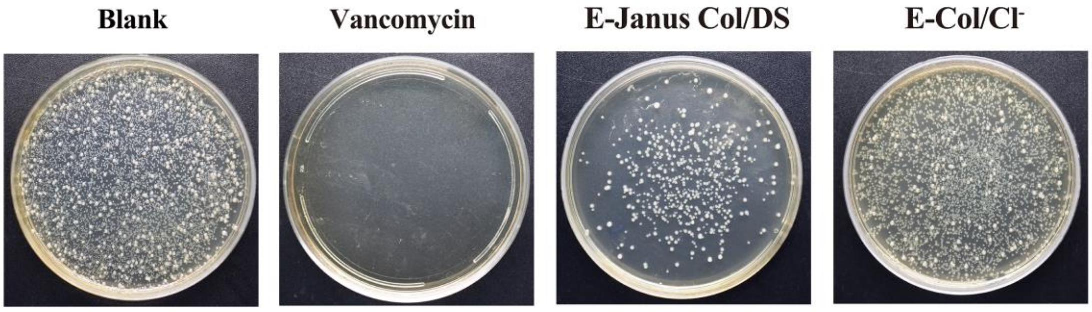

Fig. S16. Colony count of S. aureus colonies after 18 h of incubation with different samples.

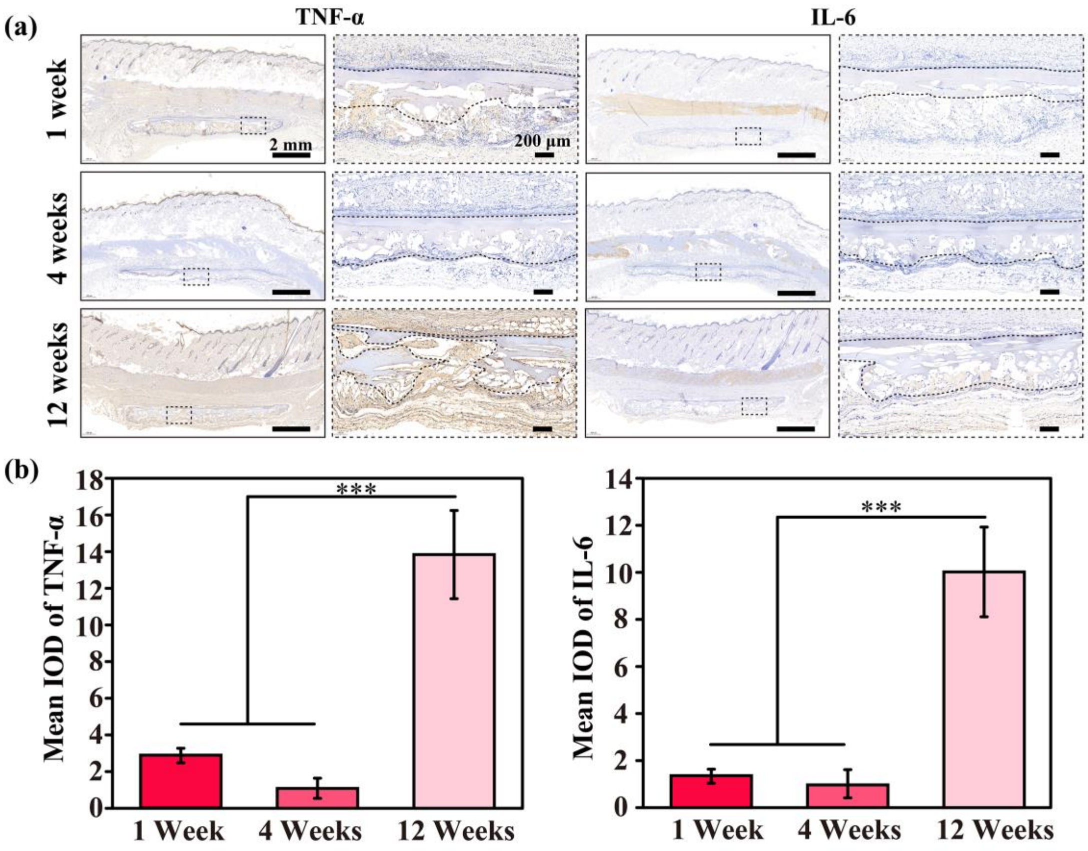

Fig. S17. (a) Representative immunohistochemistry (IHC) staining of typical inflammatory-related factors after implantation for 1, 4 and 12 weeks and (b) semi-quantification of positively stained cells, indicating Janus porous film did not show severe inflammatory at the initial stage (within 4 weeks) of implantation, and the expression of TNF-α and IL-6 was low. After 12 weeks of implantation, the film showed significant degradation and partial positive expression of TNF-α and IL-6, which is generally considered to be the normal immune response process of implant materials in vivo.[8] (n=12), three areas of 4 slice replicates in each cohort. ***$p < 0.001$. All data are presented as mean ± SD. One-way ANOVA was used for comparison.

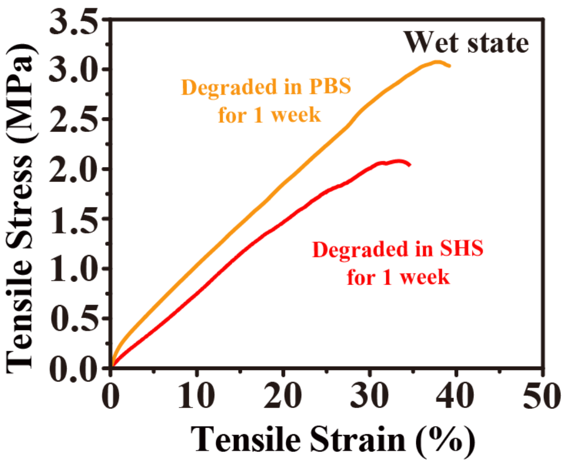

Fig. S18. The respective stress-strain curves of E-Janus Col/DS film after 1 week incubation with

simulated human salivary (i.e., SHS) or in phosphate buffered saline (i.e., PBS) at 37 ℃, indicating the Janus films incubated in SHS show slightly more significant mechanical degradation than those incubated in PBS.

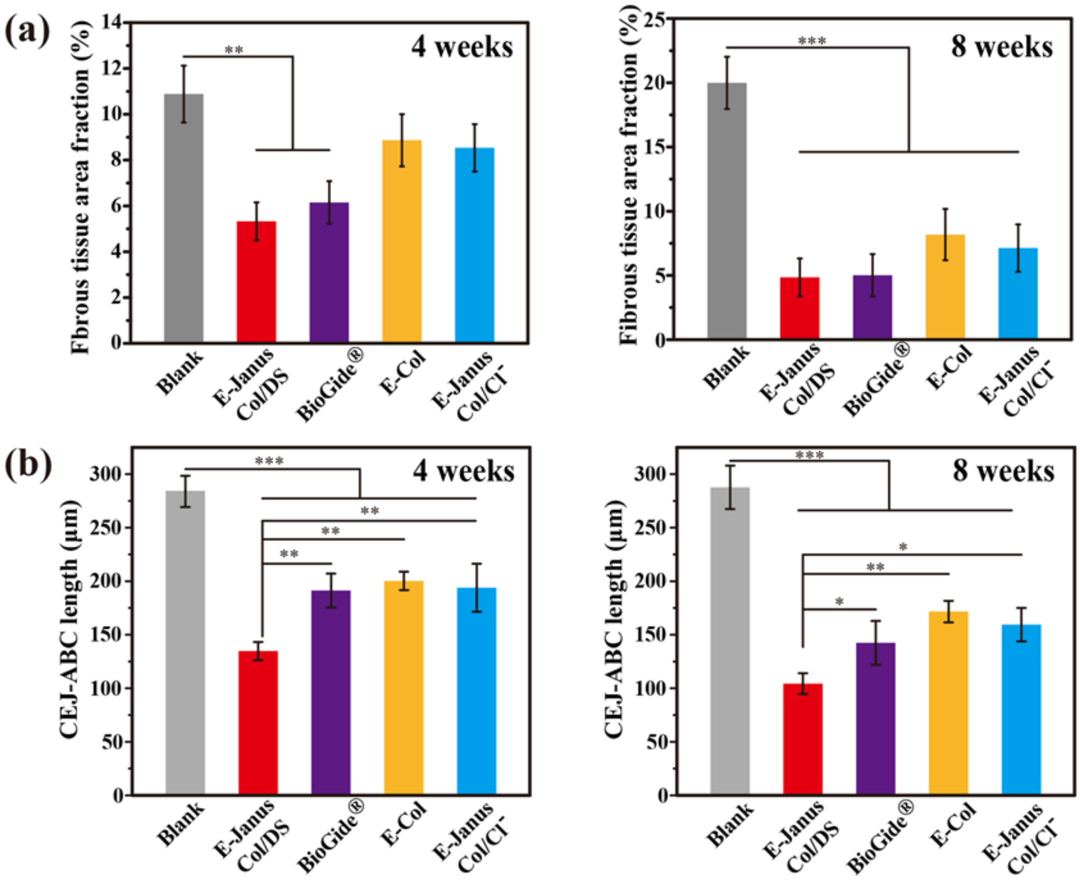

Fig. S19. Semi-quantitative analysis of histological images was performed to measure (a) the area of fibrous tissue/cells infiltrated into the alveolar bone area and (b) the distance between the alveolar bone crest (ABC) and the cementum junction (CEJ). The results showed that a larger infiltration level of fibrous tissue/cells compared to the non-implanted material group, with all implanted film groups exhibiting relatively fewer areas of fibrous tissue infiltration. Besides, the ABC-CEJ distance of the E-Janus Col/DS film implanted group decreased significantly with the extension of repair time and reached the lowest level after 8 weeks, indicating the best recovery of alveolar bone height. While there was no significant change in ABC-CEJ distance in the non-implanted material group. (n=12), three areas of 4 H&E slice replicates in each cohort. (*$p < 0.05$, **$p < 0.01$ and ***$p < 0.001$; All data are presented as mean ± SD. One-way ANOVA was used for comparison).

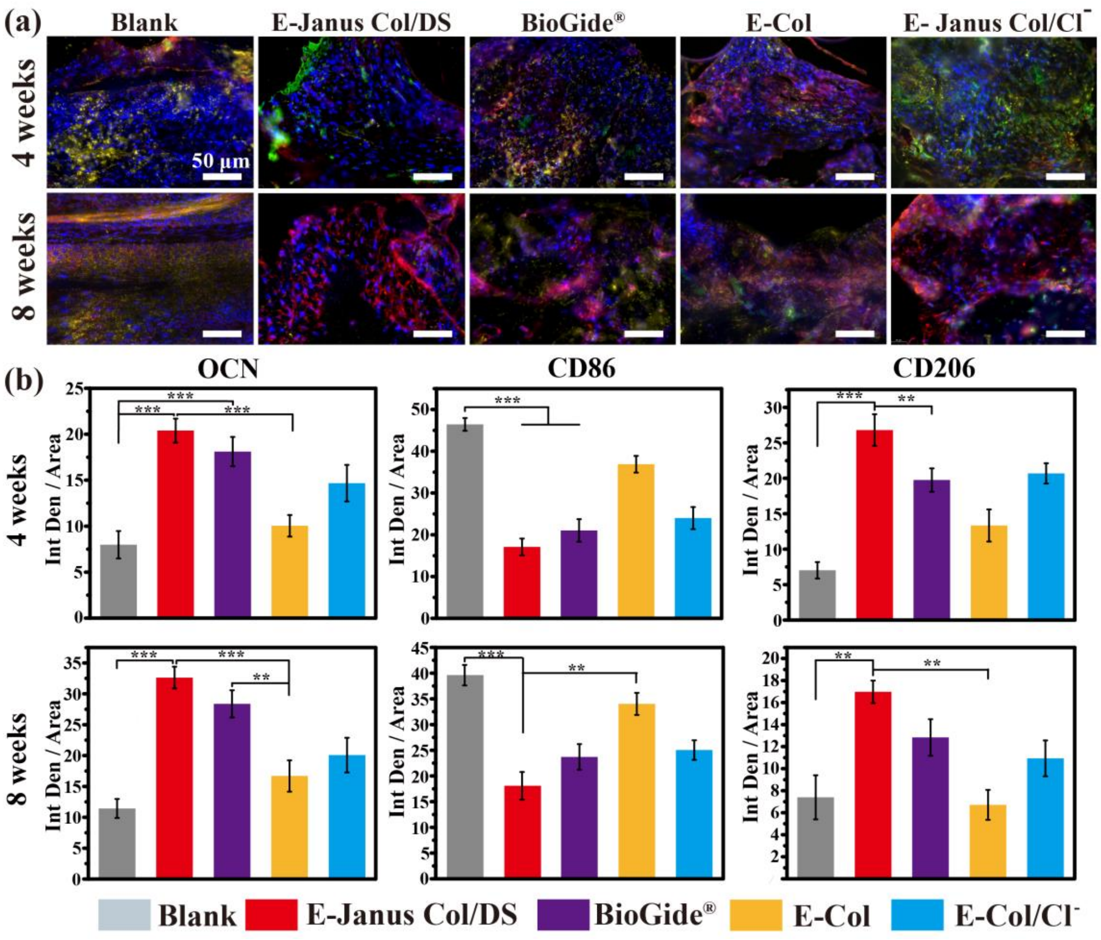

Fig. S20. (a) Representative immunofluorescence staining images of OCN (red, a marker for osteoblasts), CD86 (yellow, a marker for M1 macrophages), CD206 (green, a marker for M2 macrophages). (b) Semi-quantification of positively stained cells after implantation for 4 and 8 weeks. (n=12), three areas of 4 slice replicates in each cohort. ***$p < 0.001$. All data are presented as mean ± SD. One-way ANOVA was used for comparison.

Supplementary Movies

Movie S1. The kinetic process of collagen electro-assembly in the electrolyte absence of NaCl.

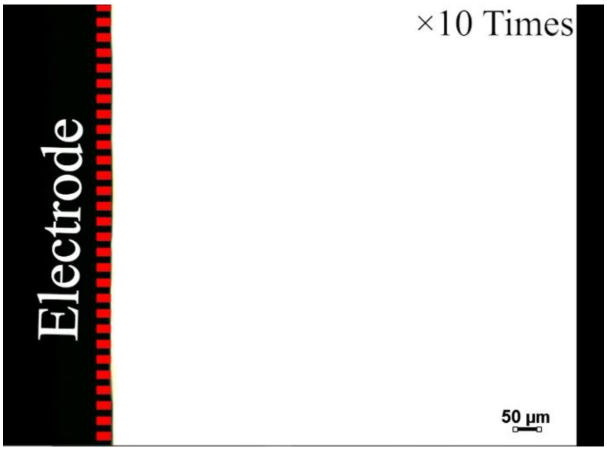

Movie S2. The kinetic process of collagen electro-assembly in the electrolyte presence of NaCl.

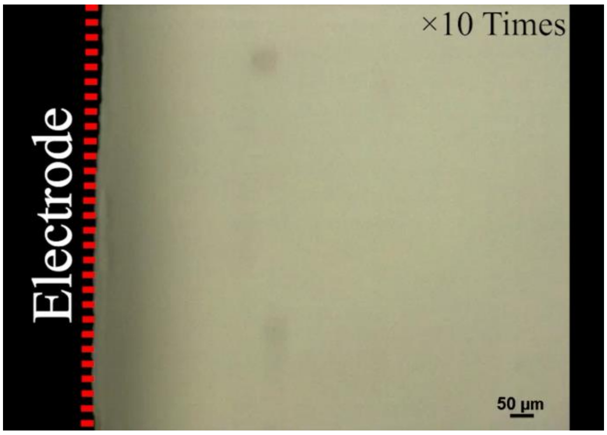

Reference

[1] a)Y. Liu, B. Zhang, K. M. Gray, Y. Cheng, E. Kim, G. W. Rubloff, W. E. Bentley, Q. Wang, G. F. Payne, *Soft Matter* **2013**, 9, 2703; b)Y. Cheng, X. Luo, J. Betz, S. Buckhout-White, O. Bekdash, G. F. Payne, W. E. Bentley, G. W. Rubloff, *Soft Matter* **2010**, 6, 3177.

[2] Q. L. Loh, C. Choong, *Tissue Eng. Part B Rev.* **2013**, 19, 485.

[3] J. Ruan, X. Wang, Z. Yu, Z. Wang, Q. Xie, D. Zhang, Y. Huang, H. Zhou, X. Bi, C. Xiao, P. Gu, X. Fan, *Adv. Funct. Mater.* **2016**, 26, 1085.

[4] J. Xue, M. He, H. Liu, Y. Niu, A. Crawford, P. D. Coates, D. Chen, R. Shi, L. Zhang, *Biomaterials* **2014**, 35, 9395.

[5] a)M. Zupanc, T. Kosjek, M. Petkovšek, M. Dular, B. Kompare, B. Širok, Ž. Blažeka, E. Heath, *Ultrason. Sonochem.* **2013**, 20, 1104; b)Q. Wei, T. Fu, L. Lei, H. Liu, Y. Zhang, S. Ma, F. Zhou, *Friction* **2023**, 11, 410.

[6] Y. Sun, Q. Zhou, Y. Du, J. Sun, W. Bi, W. Liu, R. Li, X. Wu, F. Yang, L. Song, N. Li, W. Cui, Y. Yu, *Small* **2022**, 18, e2201656.

[7] a)T. Abe, G. Hajishengallis, *J. Immunol. Methods* **2013**, 394, 49; b)J. Lin, L. Bi, X. Yu, T. Kawai, A. Taubman Martin, B. Shen, X. Han, *Infect. Immun.* **2014**, 82, 4127.
[8] a)Y. R. Cai, J. M. Guo, C. Chen, C. X. Yao, S. M. Chung, J. M. Yao, I. S. Lee, X. D. Kong, *Mat. Sci. Eng. C-Mater.* **2017**, 70, 148; b)Y. Y. Wang, R. Shi, P. Gong, J. D. Li, J. Li, D. T. Ao, P. Wang, Y. Yang, Y. Man, Y. L. Qu, *J. Bioact. Compat. Pol.* **2012**, 27, 122.
